# Challenge of Bovine Foot Skin Fibroblasts With Digital Dermatitis Treponemes Identifies Distinct Pathogenic Mechanisms

**DOI:** 10.3389/fcimb.2020.538591

**Published:** 2021-01-08

**Authors:** Kerry Newbrook, Stuart D. Carter, Hayley Crosby-Durrani, Nicholas J. Evans

**Affiliations:** Department of Infection Biology, Institute of Infection and Global Health, University of Liverpool, Liverpool, United Kingdom

**Keywords:** treponemes, bovine digital dermatitis, pathogenic mechanisms, fibroblasts, RNA-Seq

## Abstract

Bovine digital dermatitis (BDD) is a common infectious disease of digital skin in cattle and an important cause of lameness worldwide, with limited treatment options. It is of increasing global concern for both animal welfare and food security, imposing a large economic burden on cattle farming industries each year. A polytreponemal etiology has been consistently identified, with three key phylogroups implicated globally: *Treponema medium, Treponema phagedenis*, and *Treponema pedis.* Pathogenic mechanisms which might enable targeted treatment/therapeutic development are poorly defined. This study used RNA sequencing to determine global differential mRNA expression in primary bovine foot skin fibroblasts following challenge with three representative BDD treponemes and a commensal treponeme, *Treponema ruminis*. A pro-inflammatory response was elicited by the BDD treponemes, mediated through *IL-8/IL-17* signaling. Unexpectedly, the three BDD treponemes elicited distinct mechanisms of pathogenesis. *T. phagedenis* and *T. pedis* increased abundance of mRNA transcripts associated with apoptosis, while *T. medium* and *T. pedis* increased transcripts involved in actin rearrangement and loss of cell adhesion, likely promoting tissue invasion. The upregulation of antimicrobial peptide precursor, DEFB123, by *T. phagedenis* spirochaetes may present a microbial ecological advantage to all treponemes within BDD infected tissue, explaining their dominance within lesions. A commensal, *T. ruminis*, significantly dysregulated over three times the number of host mRNA transcripts compared to BDD treponemes, implying BDD treponemes, akin to the syphilis pathogen (*Treponema pallidum*), have evolved as “stealth pathogens” which avoid triggering substantial host immune/inflammatory responses to enable persistence and tissue invasion. Immunohistochemistry demonstrated increased IL-6, IL-8, RND1, and CFB protein expression in BDD lesions, confirming *in vitro* fibroblast observations and highlighting the system’s value in modeling BDD pathogenesis. Several unique shared gene targets were identified, particularly *RGS16*, *GRO1*, *MAFF*, and *ZC3H12A*. The three key BDD *Treponema* phylogroups elicited both distinct and shared pathogenic mechanisms in bovine foot skin; upregulating inflammation whilst simultaneously suppressing adaptive immunity. The novel gene targets identified here should enable future vaccine/therapeutic approaches.

## Introduction

Bovine digital dermatitis (BDD) is an inflammatory infectious disease of the foot skin of dairy and beef cattle (Blowey and Sharp, 1988; Sullivan et al., 2013). It is characterized by painful, focally inflamed, ulcerative lesions between the heel bulbs on the hind feet (Blowey and Sharp, 1988). BDD is one of the most important causes of severe cattle lameness and is of increasing global concern for both animal welfare and food security due to its significant negative impacts on milk yield, reproductive performance and production efficiency (Green et al., 2002; Bicalho et al., 2007; Bruijnis et al., 2012; Relun et al., 2013; Gomez et al., 2015; Randall et al., 2015). Now widely endemic across Europe and Northern America and reported in nearly all countries with dairy cattle industries, BDD has an economic burden estimated at $190 million per year in the USA alone (Losinger, 2006).

BDD is widely considered to have an infectious polytreponemal etiology (Stamm et al., 2002; Evans et al., 2008; Evans et al., 2009a; Klitgaard et al., 2013; Zinicola et al., 2015). Highly motile, helical, anaerobic spirochaetes of the *Treponema* genus are the only microorganisms consistently identified within BDD lesions, while not present within healthy bovine foot skin tissue (Döpfer et al., 1997; Evans et al., 2009a). Recent development of a BDD infection model further implicates a treponemal aetiology (Gomez et al., 2012). Three particular *Treponema* phylogroups have been consistently isolated from BDD lesions in the UK and USA and are implicated globally; *Treponema medium* phylogroup, *Treponema phagedenis* phylogroup and *Treponema pedis* (Stamm et al., 2002; Evans et al., 2008; Evans et al., 2009a; Klitgaard et al., 2013; Zinicola et al., 2015).

With no vaccines available, topical antibiotics and whole-herd footbaths are the mainstay of current treatments for BDD; however, lesions frequently recur (Blowey and Sharp, 1988; Berry et al., 2010; Logue et al., 2012). To facilitate development of novel, efficacious vaccines/therapeutics against BDD, we require greater understanding of the pathogenic mechanisms employed by BDD treponemes. Bovine foot skin fibroblast cells are a predominant cell lineage within dermal skin tissue and have been identified as a useful model to investigate BDD pathogenesis (Evans et al., 2014). It is now important to consider the role each BDD *Treponema* phylogroup contributes within infected tissues by using a global RNA sequencing approach to identify *de novo* host targets to enable therapeutic interventions.

This study investigated the hypothesis that three globally predominant *Treponema* phylogroups associated with BDD lesions, *T. medium* phylogroup, *T. phagedenis* phylogroup, and *T. pedis*, implement common mechanisms of pathogenesis within cells of bovine foot skin tissues during infection. Using a next generation RNA sequencing (RNA-Seq) approach, the dysregulation of global mRNA expression was investigated in primary bovine foot skin fibroblasts following challenge with representative strains of the three predominant BDD *Treponema* phylogroups. Resulting transcriptome profiles were compared to that of a commensal gastrointestinal treponeme, *Treponema ruminis* (Evans et al., 2011; Newbrook et al., 2017), to identify pathogenic signatures. Subsequently we investigate four of the most highly dysregulated mRNA targets (*IL-8, IL-6*, *RND1*, and *CFB*) for the localization of their encoded proteins within BDD lesions *in vivo* by immunohistochemistry to validate these findings.

## Materials and Methods

### Isolation and Subculture of Primary Bovine Foot Skin Fibroblasts

Primary bovine dermal fibroblast cells were isolated from visibly healthy bovine foot skin tissue as previously described (Evans et al., 2014). Isolated cells were seeded into 25 cm^3^ tissue culture flasks at 2 × 10^4^ viable cells per ml in growth media. Williams’ medium E (WME; Sigma-Aldrich, Poole, UK) was supplemented with 100 µg/ml neomycin (Sigma), 50 µg/ml gentamycin (Sigma), 20% (v/v) foetal bovine serum (Gibco™ by Thermo Fisher Scientific, Loughborough, UK), 2 mM L-glutamine (Gibco™), 2.5 µg/ml Fungizone^®^ Antimycotic (Gibco™), 10 ng/ml human recombinant epidermal growth factor (Gibco™). Cultures were maintained within a humidified incubator (37°C, 5% CO_2_) to passage eight with subculture (Evans et al., 2014).

### RNA Extraction, Quantification and Quality Control

Cell monolayers were harvested for total RNA extraction at 80% confluence by detachment (37°C, 5 min) with 0.025% (w/v) trypsin-EDTA (Gibco™). Total RNA was extracted from cells using a RNeasy^®^ Plus Mini Kit (Qiagen, Manchester, UK) and quantified with a Qubit^®^ RNA Broad-Range Assay Kit (Thermo) and Qubit^®^ 2.0 Fluorometer. RNA integrity was assessed with a Eukaryote Total RNA 6000 Nano Electophoretic Assay and 2100 Bioanalyser (Agilent Technologies Inc, Santa Clara, USA). RNA purity was measured by A260:A280 and A260:A230 ratios using a NanoDrop™ ND-2000 spectrophotometer (Thermo).

### First-Strand cDNA Synthesis

Total RNA (500 ng) was reverse transcribed to synthesize first-strand cDNA using 0.5 µg/µl oligo(dT)_12–18_ primers and SuperScript^®^ III Reverse Transcriptase (Thermo), according to manufacturer instructions. Representative reverse transcription negative (RT^−^) controls were prepared in parallel to cDNA preparations, with RNase-free water replacing reverse transcriptase, to detect contaminating gDNA by RT-PCR.

### Reverse Transcription PCR

The involucrin gene, expressed by terminally differentiated keratinocytes, was amplified from cDNA of bovine cells by RT-PCR and analyzed (Evans et al., 2014) alongside a positive control, glyceraldehyde 3-phosphate dehydrogenase (*GAPDH*).

### Immunofluorescent Labeling

IF labeling was performed on newly isolated cells to detect expression of two abundant epithelial cellular proteins, vimentin, and pan cytokeratin, for characterization. Vimentin is a key cytoskeletal intermediate filament protein of mesenchymal cells including fibroblasts, while acidic and basic cytokeratins are expressed during terminal differentiation of epidermal keratinocytes. Labeling was performed at room temperature and washes performed using Dulbecco’s phosphate buffered saline (calcium-magnesium free; DPBS-CMF). Cell monolayers were washed then fixed for 30 min with 4% (w/v) formaldehyde (Thermo). Autofluorescence was quenched (30 min) with 50 mM ammonium chloride (Sigma). Monolayers were blocked (1 h) with IF block [DPBS-CMF, 10% (v/v) donkey serum (Sigma), 1% (v/v) Triton™ X-100 (Sigma)] and incubated (1 h) with rabbit anti-vimentin polyclonal antibody (1:200) and mouse anti-pan cytokeratin monoclonal antibody (1:200) (Abcam^®^, Cambridge, UK) to simultaneously detect vimentin and pan cytokeratin [56.5/50/48/40 kDa (acidic) and 65–67/64/59/58/56/52 kDa (basic)]. Unbound antibodies were removed with IF wash [DPBS-CMF, 1% (v/v) donkey serum, 0.1% (v/v) Triton™ X-100] and bound antibodies detected by 2 h incubation with corresponding polyclonal antibodies [tetramethylrhodamine isothiocyanate (TRITC)-conjugated donkey anti-rabbit IgG (H&L) and Alexa Fluor^®^ 488-conjugated donkey anti-mouse IgG (H&L)] (Abcam), diluted 1:200 and 1:300 respectively. Fluorescence visualized with an Olympus CK40 inverted phase contrast microscope (Carl Zeiss Ltd., Cambridge, UK) and images analyzed using ZEN lite 2 (Carl Zeiss Ltd).

### Immunohistochemistry

IHC was performed on bovine foot skin from cows without BDD lesions (lesion stage M0, n = 3), acute BDD lesions (lesion stage M2, n = 3) and chronic BDD lesions (lesion stage M4, n = 3) (Döpfer et al., 1997) to detect expression of CFB, RND1, IL-6 and IL-8 *in vivo*. Sections (4 µm) from archived paraffin embedded tissue blocks were mounted onto glass slides, deparaffinized and antigen retrieval carried out using DAKO EnVision™ FLEX target retrieval solution high pH (Agilent) at 95°C for 25 min. Using DAKO Autostainer Link 48 (Agilent), sections were washed, and endogenous peroxidase blocked, prior to incubation (20 min) with corresponding primary antibodies at optimal dilutions for bovine skin (**Table 1**). IHC antibodies were each tested and their labeling optimized on appropriate tissues before use. Both negative antibody (no primary antibody) and internal labeling controls were used to confirm positive labeling and determine background staining. Sections were incubated (20 min) with label polymer, DAKO EnVision™ FLEX/HRP and bound antigen subsequently detected using DAKO EnVision™ FLEX DAB + chromogen. Sections were counterstained with hematoxylin. IHC using an anti-DD treponeme antibody (**Table 1**) was also performed, as previously described (Crosby-Durrani et al., 2016), to confirm the presence of DD treponemes in the sections.

**Table 1:** Primary antibodies used for IHC on bovine foot skin tissues.

Target	Antibody	Dilution	Source
CFB	Rabbit anti-CFB polyclonal antibody	1:100	Invitrogen (Thermo) (Cat # PA5-51640)
RND1	Rabbit anti-RND1 polyclonal antibody	1:50	Abcam (Cat # ab222331)
IL-6	Rabbit anti-IL-6 polyclonal antibody	1:100	Abcam (Cat # ab6672)
IL-8	Mouse anti-IL-8 monoclonal antibody (clone 8M6)	1:100	Abcam (Cat # ab34100)
Treponeme	Rabbit anti-DD associated treponeme polyclonal antibody	1:4,000	In house (Evans et al., 2009a)

### Treponeme Culture

*T. medium* phylogroup strain T19, *T. phagedenis* phylogroup T320A and *T. pedis* strain T3552B^T^ were previously isolated from BDD lesion biopsies taken from foot skin tissue of Holstein–Friesian dairy cattle in Merseyside, UK (Evans et al., 2008). *T. ruminis* strain Ru1^T^ was previously isolated from rumen contents of a Holstein–Friesian bull in Cheshire, UK (Evans et al., 2011). Treponeme cultures maintained as previously described (Evans et al., 2011; Klitgaard et al., 2013).

### Preparation of Treponeme Sonicates

Treponeme sonicates were prepared from late exponential growth phase cultures (Evans et al., 2014). Sonication was performed on ice for 4 min (45% amplitude) using continuous, alternating cycles (10 s) of sonication and resting (Sonics VibraCell™ VCX130; VWR^®^, Lutterworth, UK). Sonicates were quantified with a Qubit^®^ Protein Assay Kit and Qubit^®^ 2.0 Fluorometer (Thermo) and diluted in control medium [WME, 2 mM L-glutamine] for use.

### Co-Incubation of Bovine Fibroblasts With Treponeme Sonicates

Primary bovine foot skin fibroblasts (1 × 10^5^) were seeded into 25 cm^3^ tissue culture flasks and maintained to 80% confluence. Monolayers were washed with 1× HBSS and co-incubated (3 ml) for 6 h with control media, 10 µg/ml purified LPS from *S.* Typhimurium (Sigma) (positive control stimulant) or 10 µg/ml treponeme sonicate (either *T. medium*, *T. phagedenis*, *T. pedis* or *T. ruminis*) in a humidified incubator (5% CO_2_, 37°C). A 6-h co-incubation time point was determined most optimal for detecting mRNA expression in treponeme-stimulated fibroblasts based on previous preliminary data (Evans et al., 2014) and is within-range of that of previous spirochaete co-incubation studies (Ebnet et al., 1997; Zuerner et al., 2007; Evans et al., 2014). Each treatment performed in triplicate technical and experimental replicates. Treatments subsequently removed by washing and monolayers harvested for RNA extraction.

### RNA Sequencing

Total RNA of highest quality and quantity (**Table S2**), representing triplicate experimental replicates of each challenge group (n = 3), were submitted for RNA-Seq at the Centre for Genomic Research (University of Liverpool, UK). An additional technical replicate was analyzed for one of the experimental replicates in both *T. phagedenis* and *T. ruminis* challenge groups (n = 4).

Total RNA (1,000 ng) was depleted of rRNA using a Ribo-Zero™ Gold rRNA removal (human/mouse/rat) kit (Illumina^®^, San Diego, USA). RNA-Seq (cDNA) libraries were prepared using NEBNext^®^ Ultra™ Directional RNA Library Prep Kit (NEB, Hertfordshire, UK) and purified using AMPure XP beads. Libraries were diluted (1:4) in 0.1× Tris EDTA buffer and quantified using a Qubit^®^ dsDNA High Sensitivity Assay Kit (Thermo) and Qubit^®^ 2.0 Fluorometer. Size distribution was assessed using an Agilent High Sensitivity DNA Kit and 2100 Bioanalyser (Agilent), according to manufacturer instructions. Libraries were pooled in equimolar amounts (n = 10, two pools), quality re-assessed and quantified by qPCR (LightCycler^®^ LC48011) with a KAPA Library Quantification Kit (Illumina^®^). PhiX Control V3 (Illumina^®^) used as positive internal control library. Denatured template cDNA was loaded onto the flow cell for clustering using a HiSeq^®^ 3000/4000 Paired-End Cluster Kit with cBot™ Cluster Generation System (Illumina^®^). Libraries were sequenced on a HiSeq™4000 (Illumina^®^) using version 1 chemistry to generate 2 × 150 bp paired-end reads. An average of 66,911,863 (range 44,401,794 to 102,256,288) raw sequenced reads was obtained per sample (**Figure S5**). FastQ files were trimmed to exclude reads matching Illumina adaptor sequences of ≥3 bp at 3′ end using option –O 3 in Cutadapt V1.2.1 (Martin, 2011). Sickle V1.200^1^ was used to trim poor quality reads (minimum window quality score 20) and those below 10 bp. On average, 66,333,535 (range 44,104,369 to 101,608,258) trimmed sequenced reads proceeded to further analysis (**Figure S5**).

### RNA Sequencing Analysis

A reference-based assembly was used for transcriptional profiling, using an RNA-Seq analysis pipeline (**Figure S6**) (Trapnell et al., 2012), implemented using Galaxy Platform’s public server^2^ (Afgan et al., 2016). Trimmed reads were mapped to the *Bos Taurus* genome (UMD 3.1.1/bosTau8; GenBank Accession GCA_000003055.5) using TopHat2 (Kim et al., 2013), with average mapping rate of 85.74% (range 78.00–90.30%). Default mapping parameters were used, allowing two read mismatches, 160 ± 60 bp mean inner distance between mate pairs and maximum one read alignment to genome. The percentage of average aligned reads per mate pair is given for each challenge group in **Table S3**. Binary alignment/map files were assessed for accurate read mapping using Interactive Genome Viewer (Robinson et al., 2011). Mapped reads were assembled into transcripts using Cufflinks and a master transcriptome generated by Cuffmerge, using *Bos Taurus* genome (UMD 3.1.1/bosTau8) as reference (Trapnell et al., 2010). Differential fibroblast mRNA expression profiles (mean FPKM) were generated, compared to the media control with Cuffdiff (Trapnell et al., 2010); using the master transcriptome assembly and mapped read files for corresponding replicates of challenge and control groups. Default parameters used included geometric library normalization, pooled dispersion estimation, multi-read correct, bias correction and false discovery rate (FDR) of 0.05. mRNA transcripts with an adjusted *p (q* or FDR) value ≤ 0.05 were considered to have statistically significantly different abundances, with a log2 fold change cut-off (≥1 and ≤ −1) used to define biological significance; these criteria were implemented for significance exclusion in all downstream analyses. Venn diagrams prepared with Venny V2.0^3^ using Ensembl gene identifiers of mRNA transcripts with significantly different abundances. For simplicity, LPS control data was not included for comparison. Abundance heat maps were generated by Genesis V1.8.1 (Sturn et al., 2002) from log2 fold change values corresponding to 20 greatest increased/decreased mRNA transcripts in each treatment group and scales adjusted within limits of observed transcript abundance.

### Ingenuity Pathway Analysis

Comparison of significantly enriched canonical pathways, diseases, biological functions and upstream regulators was performed using Ingenuity Pathway Analysis (IPA) software (Qiagen, Denmark). Differentially expressed mRNA transcripts across treatment groups were assigned stable bovine gene identifiers by Cuffdiff, using the reference genome (UMD 3.1.1 *Bos Taurus*) (Aken et al., 2017) to resolve transcripts with multiple identifiers (**Table S4**). Bovine gene identifiers were converted to orthologous human equivalents using Ensembl (Kinsella et al., 2011; Aken et al., 2017) and uploaded into IPA alongside corresponding log2 fold change and *p* and *q* values. Where bovine transcripts mapped to multiple human orthologs (~3.83% genes/treatment group), reciprocal nucleotide BLASTs were used to predict gene orthology, resolving 51.85% cases with remaining unresolved cases excluded. Core IPA was performed applying previously described significance parameters, to produce “analysis-ready” molecules (LPS: n = 207, T19: n = 48, T320A: n = 370, T3552B^T^: n = 365, Ru1: n = 1372) and subsequently analyzed by comparative IPA.

### Variance of Experimental Replicates

Cuffnorm (Trapnell et al., 2010) implemented in Galaxy to provide normalized transcript abundance profiles for individual replicates using corresponding mapped read files and master transcriptome assembly. PCA performed on normalized FPKM expression data for each replicate using Genesis V1.8.1 (Sturn et al., 2002) to establish replicate quality and variation across treatment groups. HCL analysis, using the average linkage WPGMA method, performed on normalized FPKM expression data for each replicate in Genesis V1.8.1 (Sturn et al., 2002) to establish replicate clustering patterns.

### Linear Regression Analysis

Linear regression performed in Graphpad Prism V5 (San Diego, California, USA) by pairwise treatment group comparisons of log2 fold change abundance data (20 most significantly increased/decreased transcripts). Linear correlation coefficients (r) used to determine relationships of positive correlation (r > 0), negative correlation (r < 0) and no correlation (r = 0) (significance, *p* ≤ 0.05).

## Results

An *in vitro* skin cell model was used to model and dissect the complex microbial environment of the bovine hoof and specifically elucidate the interactions and individual pathogenic contributions of three BDD *Treponema* phylogroups with bovine foot skin fibroblast cells, as the predominant cell lineage of dermal skin, using RNA-Seq.

### Isolation and Characterization of Bovine Foot Skin Fibroblast Cells

**Figure 1:**
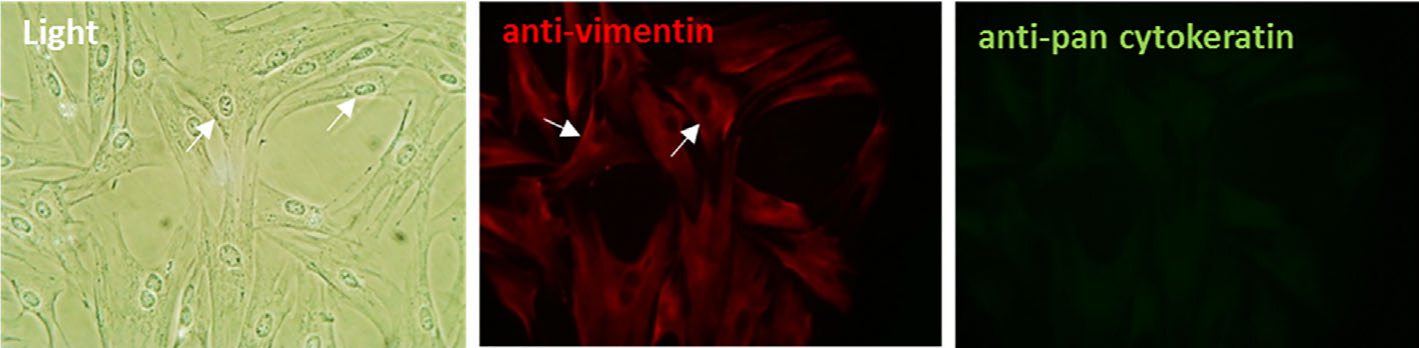
Characterization of primary bovine foot skin fibroblast cells. Primary fibroblast cells demonstrated prominent cytoplasmic projections (arrows) and the elongated spindle-shaped morphology which is typical of fibroblasts in culture. Following initial subculture, cells demonstrated positive immunofluorescence labeling for the key intermediate filament protein of fibroblasts, vimentin (anti-vimentin; red) and negative labeling for several acidic (56.5/50/48/40 kDa) and basic (65–67/64/59/58/56/52 kDa) cytokeratins (anti-pan cytokeratin; green), which are intermediate filament proteins typically expressed by epidermal keratinocytes. Data representative of three independent experimental replicates. Scale, ×20 magnification.

Primary bovine fibroblast cells were successfully isolated and subcultured from healthy, full-thickness dermal foot skin tissue. Following subculture, cells underwent characterization. By phase contrast microscopy, cells exhibited prominent cytoplasmic projections and the elongated, spindle-shaped morphology characteristic of fibroblasts (**Figure 1**). Immunofluorescence (IF) labeling confirmed expression of fibroblast intermediate filament protein, vimentin (**Figure 1**). Cells demonstrated no labeling for acidic (56.5/50/48/40 kDa) and basic (65–67/64/59/58/56/52 kDa) cytokeratins typically expressed by epidermal keratinocytes (**Figure 1**) and reverse transcription PCR (RT-PCR) further confirmed lack of expression of a typical epidermal cell marker, involucrin (**Figure S1**).

### BDD *Treponema* Phylogroups Elicit Distinct Changes in Global mRNA Expression of Bovine Foot Skin Fibroblast Cells

Transcriptome profiles of primary bovine foot skin fibroblast cells were generated by RNA-Seq and identified significant changes in global mRNA expression following 6 h challenge (Evans et al., 2014), with a minimum of three experimental replicates, with sonicated preparations of *T. medium* phylogroup strain T19 (n = 3), *T. phagedenis* phylogroup strain T320A (n = 4), *T. pedis* strain T3552B^T^ (n = 3), *T. ruminis* strain Ru1^T^ (n = 4) and lipopolysaccharide (LPS) (positive control, n = 3) from *Salmonella enterica* serotype Typhimurium (**Table 2**). An average overall combined read mapping rate of 85.74% (78.00–90.30%) was achieved (**Table S3**), with average read depths of 27,693,481 (media), 26,783,104 (LPS control), 34,342,019 (T19), 24,471,173 (T320A), 27,498,649 (T3552B^T^), and 29,489,722 (Ru1) respectively.

**Table 2:** Total number of transcripts with significantly different expression in bovine foot skin fibroblasts challenged with treponemes.

Challenge group	Significantly differentially expressed mRNA transcripts (q ≤ 0.05)			Significantly differentially expressed mRNA transcripts (q ≤ 0.05, log2 fold change ≥ 1 or ≤ −1)	
	All log2 fold change	Log2 fold change ≥ 2 or ≤ −2	Log2 fold change ≥ 1 or ≤ −1	Increased	Decreased
*S.* Typhimurium	817	41	246	227	19
*T. medium* (T19)	88	22	58	54	4
*T. phagedenis* (T320A)	1,338	15	398	134	264
*T. pedis* (T3552B^T^)	670	39	395	38	357
*T. ruminis* (Ru1^T^)	3,185	61	1,469	763	706

RNA-Seq was used to determine total numbers of fibroblast mRNA transcripts with significantly different expression for each 6-h challenge group (T. medium phylogroup strain T19 sonicate, T. phagedenis phylogroup strain T320A sonicate, T. pedis strain T3552B^T^ sonicate, T. ruminis strain Ru1^T^ sonicate or S. Typhimurium) compared to the media control group. Significance was defined as an FDR-adjusted p value (q value) ≤ 0.05 and a log2 fold change ≥1 and ≤**−**1 was used as a cut-off during this study. Data represents mean differential mRNA expression of three independent experimental replicates per challenge group (n = 3), with both T. phagedenis and T. ruminis represented by an additional technical replicate (n = 4).

*T. ruminis* induced the most changes in mRNA expression across the fibroblast transcriptome, while *T. medium* was least stimulatory, with just 58 significantly differentially expressed transcripts. While most mRNA transcripts differentially increased, the majority associated with challenge by *T. phagedenis* and *T. pedis* decreased, highlighting the divergent host responses to individual treponeme phylogroups. Notably, fibroblast cells also demonstrated a substantial host response to *S.* Typhimurium LPS.

**Figure 2:**
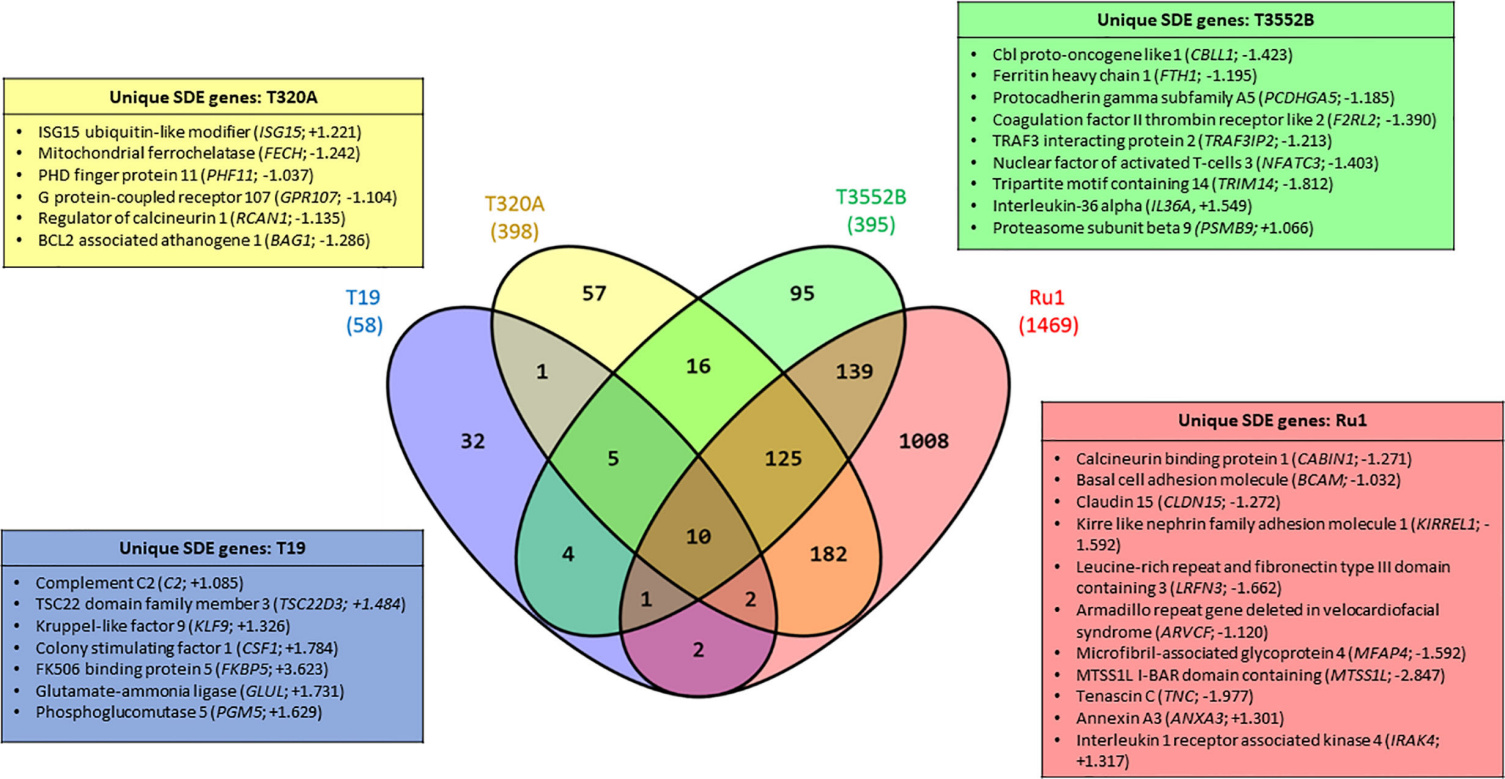
Significantly dysregulated mRNA transcripts common and unique to bovine foot skin fibroblasts challenged with pathogenic and commensal treponemes. Venn diagram illustrating the numbers of mRNA transcripts with common and unique significantly different abundances (FDR-adjusted *p value* ≤ 0.05, log2 fold change ≥1 and ≤−1) in bovine foot skin fibroblasts challenged with *T. medium* phylogroup (strain T19), *T. phagedenis* phylogroup (strain T320A), *T. pedis* (strain T3552B^T^) or *T. ruminis* (strain Ru1^T^). The total number of significantly dysregulated transcripts per challenge group is given in parentheses under the treponeme strain. A list of several key unique transcripts for each challenge group is given in corresponding colour-coded boxes, alongside their corresponding observed log2 fold changes. Data represents the mean differential mRNA expression of three independent experimental replicates per challenge group (n = 3), with both *T. phagedenis* and *T. ruminis* represented by an additional technical replicate (n = 4).

When comparing fibroblast mRNA transcripts which were commonly or uniquely dysregulated by each of the treponemes (**Figure 2**), only ten were stimulated (each increased) by all four treponeme challenge groups. These included interleukin-6 (*IL6*), complement factor B (*CFB*), nuclear factor kappa-light-chain-enhancer of activated B cells (*NF-kB*) inhibitor zeta (*Mail*), tumor necrosis factor superfamily member 15 (*TNFSF15*), cytidine deaminase (*CDA*), superoxide dismutase 2 (mitochondrial; *SOD2*) and several members of C-X-C and C-C motif chemokine subfamilies (*CXCL2*, *CXCL3*, *CXCL5*, and *CCL2*). The three pathogenic BDD treponemes (*T. medium, T. phagedenis* and *T. pedis*) commonly induced significant upregulation of a further five mRNA transcripts; regulator of G-protein signaling 16 (*RGS16*), MAF bZIP transcription factor F (*MAFF*), zinc finger CCCH-type containing 12A (*ZC3H12A*) and the pro-inflammatory chemokines, interleukin-8 (*IL-8*) and chemokine (C-X-C motif) ligand 1 (*GRO1*). Notably, all 15 of these mRNA transcripts were also significantly dysregulated by *S.* Typhimurium. While a high proportion of fibroblast mRNA transcripts were uniquely significantly dysregulated following challenge with *T. medium* and *T. ruminis* (55.17 and 68.62% respectively), the majority of significantly dysregulated transcripts resulting from *T. phagedenis* and *T. pedis* challenge (45.73 and 35.19% respectively) were shared with *T. ruminis* or all three challenge groups (~31%).

**Figure 3:**
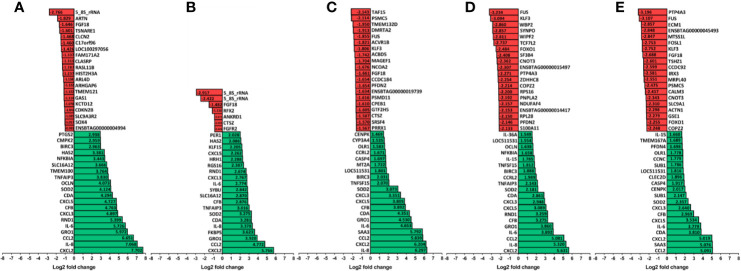
The 20 most upregulated and downregulated mRNA transcripts in bovine foot skin fibroblasts following challenge with pathogenic or commensal treponemes. Significantly dysregulated mRNA transcripts with the 20 most increased (green) and decreased (red) significant log2 fold changes (FDR-adjusted *p value ≤*0.05 and a log2 fold change ≥1 and ≤−1) in bovine foot skin fibroblasts challenged with **(A)** LPS from *S.* Typhimurium (control), **(B)** *T. medium* phylogroup (strain T19), **(C)** *T. phagedenis* phylogroup (strain T320A), **(D)** *T. pedis* (strain T3552B^T^) or **(E)** *T. ruminis* (strain Ru1^T^) are summarised. Data represents the mean differential mRNA transcript expression of three independent experimental replicates per challenge group (n = 3), with both *T. phagedenis* and *T. ruminis* represented by an additional technical replicate (n = 4). Log2 fold change values are provided within each bar for clarity and mRNA transcripts are given in abbreviated form against the corresponding bars.

**Figure 4:**
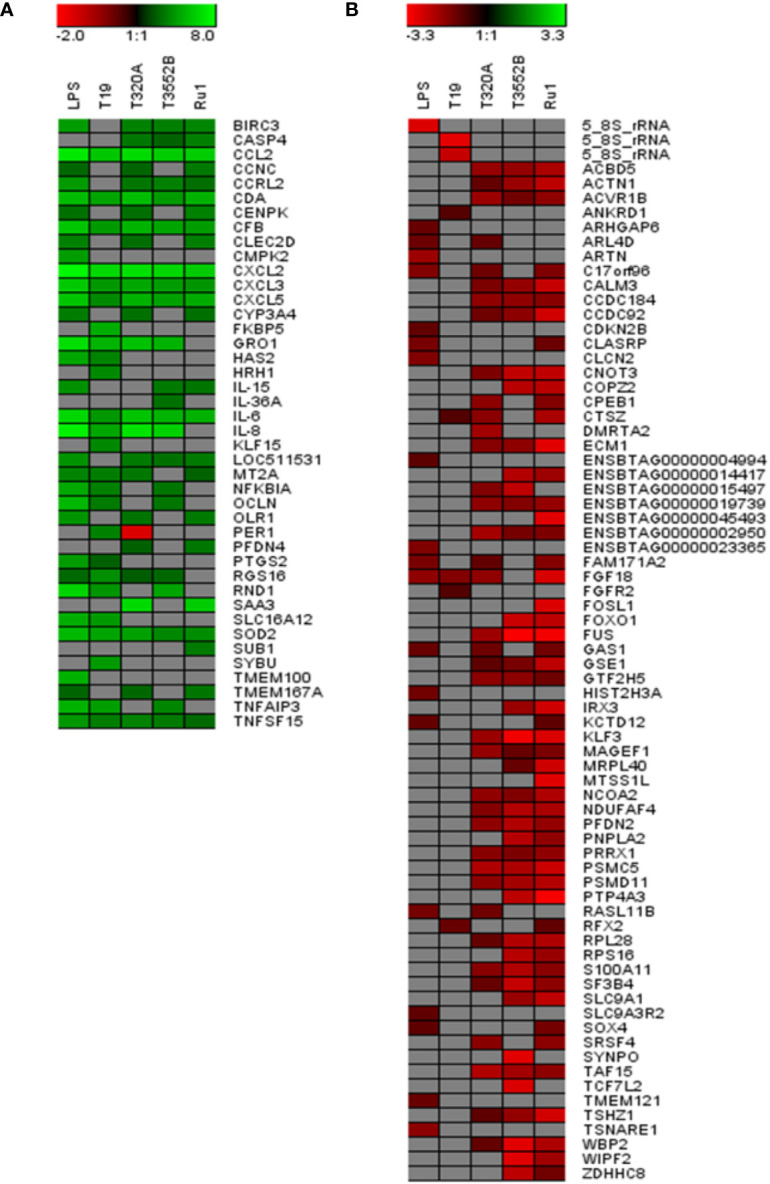
Comparison of the 20 most increased and decreased mRNA transcripts in primary bovine foot skin fibroblasts challenged with pathogenic or commensal treponemes. Heatmaps comparing the 20 most **(A)** increased and **(B)** decreased mRNA transcripts (FDR-adjusted *p* value ≤0.05 and a log2 fold change ≥1 and ≤−1) in bovine foot skin fibroblasts challenged with *T. medium* phylogroup (strain T19), *T. phagedenis* phylogroup (strain T320A), *T. pedis* (strain T3552B^T^) and *T. ruminis* (strain Ru1^T^). Transcripts are given in abbreviated form against the corresponding bars, and expression bars above each heatmap illustrate the degree of increased (green) or decreased (red) expression (log2 fold change). Gray squares indicate that no significant difference was identified compared to the media control group. Data represents the mean differential transcript expression of three independent experimental replicates per challenge group (n = 3), with both *T. phagedenis* and *T. ruminis* represented by an additional technical replicate (n = 4).

The 20 most increased and decreased fibroblast mRNA transcripts from each challenge group were summarized (**Figure 3**) and collated into heat maps (**Figure 4**) for comparison. The increased transcription of apoptotic mediators, such as caspase 4 (*CASP4*) and baculoviral IAP repeat-containing protein 3 (*BIRC3*), were consistent features of challenge with all treponemes except *T. medium*. Both *T. medium* and *T. pedis* induced significant changes in transcription of occulin (*OCLN*), Rho family GTPase 1 (*RND1*) and inflammatory mediators such as NF-*κ*B inhibitor alpha (*NFKBIA*) and TNF alpha-induced protein 3 (*TNFAIP3*). Consistent with being the least stimulatory, *T. medium* induced a significant decrease of just four mRNA transcripts; fibroblast growth factor 18 (*FGF18*), regulatory factor X2 (*RFX2*) and two 5.8S rRNAs. A particularly noticeable signature of *T. pedis* challenge was the dysregulation of fibroblast mRNA transcripts associated with actin rearrangement and cytoskeletal structure. Such candidates included four and a half LIM domains 3 (*FKL3*), nexilin F-actin binding protein (*NEXN*), cofilin-1 (*CFL1*) and Ras homolog family member G (*RHOG*). Others, such as actinin alpha 1 (*ACTN1*), synaptopodin (*SYNPO*) and WAS/WASL interacting protein family member 2 (*WIPF2*), were significantly decreased by both *T. pedis* and *T. ruminis*. Interestingly, while *T. medium* induced a significant increase in transcript abundance of period circadian clock 1 (*PER1*), *T. phagedenis* caused a decrease.

Several mRNA transcripts of the fibroblast transcriptome were found to only have detectable expression, measured as fragment per kilobase of transcripts per million mapped reads (FPKMs), within either the control or challenge group and were therefore not attributable to a log2 fold change. While the majority were non-coding RNAs, several protein-coding genes were identified. Beta-defensin 123 precursor (*DEFB123*) was expressed only in fibroblasts challenged with *T. phagedenis* sonicate (2.32 FPKM). Likewise, long-pentraxin 3 (*PTX3*) was only expressed following challenge with *T. medium* phylogroup sonicate (1.25 FPKM). No genes were identified which were only expressed within the *T pedis* sonicate-treated fibroblasts.

**Figure 5:**
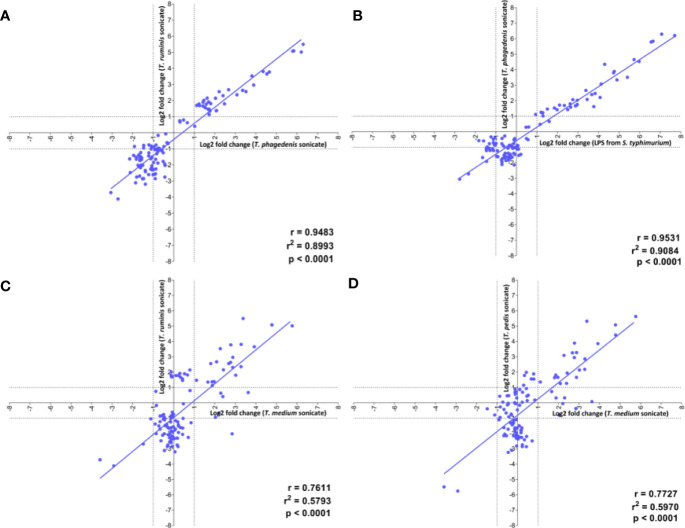
Correlation of the transcript proﬁles of primary bovine foot skin ﬁbroblasts challenged with pathogenic or commensal treponemes. Linear regression analysis was used to determine the correlation of global differential mRNA expression, represented as log2 fold change of the 20 greatest increased and decreased transcripts, between the following pairwise comparisons: **(A)** *T. phagedenis* versus *T. ruminis*, **(B)** *S. Typhimurium* versus *T. phagedenis*, **(C)** *T. medium* versus *T. ruminis*, **(D)** *T. medium* versus *T. pedis*. The regression line (gray) for each pairwise treatment comparison is shown alongside its corresponding linear correlation coefﬁcient (r), coefﬁcient of determination (r2) and p value (p), where p ≤0.05 represents statistical signiﬁcance. Data represents mean differential transcript expression of three independent experimental replicates per challenge group (n = 3), with both *T. phagedenis* and *T. ruminis* represented by an additional technical replicate (n = 4).

Linear regression analysis identified the strongest positive correlation in transcriptome profiles of fibroblasts challenged with the LPS stimulant and *T. phagedenis* (r = 0.9531) (**Figure 5B**), *T. phagedenis* and *T. ruminis* (r = 0.9483) (**Figure 5A**) and then *T. pedis* and *T. ruminis* (r = 0.9192) (**Table S1**). The weakest positive correlation was found between the profiles of *T. medium* and *T. ruminis* challenge (r = 0.7611) (**Figure 5C**) and then *T. medium* and *T. pedis* (r = 0.7727) (**Figure 5D**).

### Enriched Host Canonical Pathways and Biological Functions Following Treponeme Challenge

**Figure 6:**
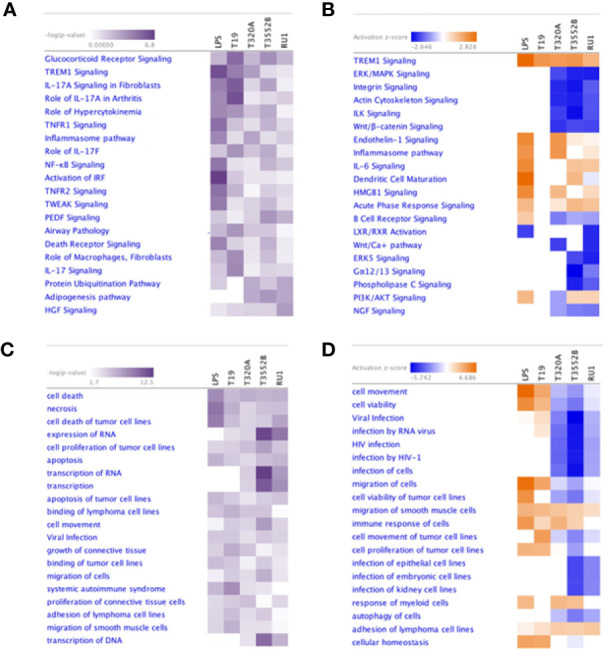
Comparison of the most significantly enriched canonical pathways, diseases and biological functions in primary bovine foot skin fibroblasts challenged with pathogenic and commensal treponemes. Ingenuity pathway analysis identified the most significantly enriched **(A)** canonical pathways and **(C)** diseases and biological functions in primary bovine foot skin fibroblasts challenged with *T. medium* phylogroup (strain T19), *T. phagedenis* phylogroup (strain T320A), *T. pedis* (strain T3552B^T^) and *T. ruminis* (strain Ru1^T^). A right-tailed Fisher Exact Test was used to calculate the probability of enrichment compared to the IPA database, denoted as –log(*p* value) with *p ≤*0.05 for statistical significance. The most activated (orange) or inhibited (blue) **(B)** canonical pathways and **(D)** diseases and biological functions were calculated with an IPA z-score algorithm. The greater the z-score beyond 2 the greater the prediction for activation, whereas the greater the z score below −2 the greater the prediction for inhibition. Unknown or undetermined activity was denoted by gray and white respectively. For simplicity, heatmaps only document the 20 most enriched canonical pathways, diseases and biological functions for each parameter.

Ingenuity pathway analysis (IPA) predicted significant enrichment of several canonical pathways, disease processes and biological functions in fibroblasts following challenge with treponemes (**Figure S2**), as summarized using comparative heat maps (**Figure 6**). Notably, TREM1 signaling was enriched in fibroblasts following challenge with all pathogenic treatments but not *T. ruminis.* Similarly, several interleukin-17 (IL-17) signaling pathways were significantly enriched across all challenge groups. Pathways associated with cell adhesion, proliferation, and cytoskeletal structure, such as integrin and actin signaling, were inhibited by *T. phagedenis, T. pedis*, and *T. ruminis* challenge. Furthermore, while B cell receptor signaling was LPS-activated, most treponeme challenges inhibited this pathway.

The host inflammatory response and infectious, immunological and inflammatory disease signatures were enriched by all challenge groups; however, both cell-mediated immune responses and hypersensitivity responses were pathogen-specific. While challenge by all groups was predicted to induce cell movement and proliferation, immune cell migration (phagocytes) and chemotaxis (leukocytes, especially neutrophils) were prominent inferred features of challenge by both *T. medium* and *T. phagedenis*. Cell viability and survival were predicted to decrease following challenge by all treatments except *T. medium* and *S.* Typhimurium. Interestingly, while necrotic and apoptotic functions were predictably increased by BDD treponeme challenge, *T. ruminis* appeared to be an inhibitor. All challenge groups except *T. pedis* were predicted to target genes associated with the activation of carbohydrate metabolism and metabolic disease, while *T. medium* was shown to favor those of energy production and nutritional disease. Interestingly, *T. phagedenis* challenge was predicted to induce a pathogenic phenotype associated with periodontal disease.

### Analysis of Variance Between Experimental Replicates

Multivariate analyses were performed to determine influence of experimental variation across the dataset. Principle component analysis (PCA) identified 82.5% dataset variation to be explained by the first principle component, with the second and third explained by a further 4.67 and 3.13% respectively (**Figure S3Ai**). Accounting for >90% dataset variation, PC1 *vs* PC2 (**Figure S3Aii**) and PC2 *vs* PC3 plots (**Figure S3Aiii**) demonstrated two distinct clusters, not related to treatments, matching the two RNA-Seq cDNA library pools thereby, suggesting an experimental batch effect. Hierarchical clustering analysis (HCA) revealed a variance pattern consistent with PCA (**Figure S3B**).

### Increased Expression of IL-6, IL-8, RND1, and CFB Proteins *In Vivo* in Bovine Foot Skin Tissues With BDD Lesions

**Figure 7:**
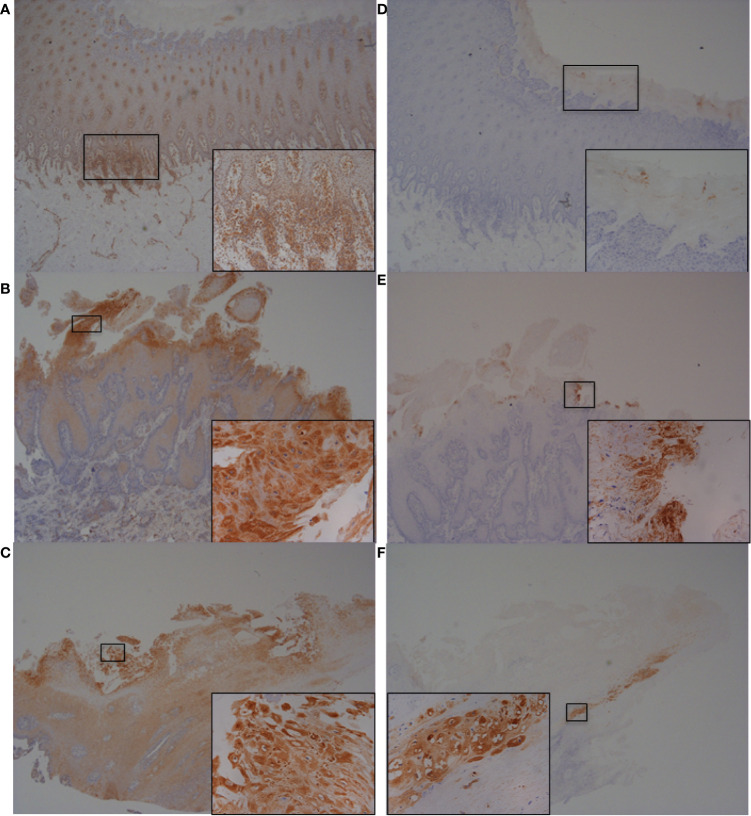
Immunohistochemistry (IHC) of CFB and RND1 on bovine foot skin from the site of BDD lesions. Low power images accompanied with high power inserts. Images shown are representatives of three independent experimental replicates (n = 3) for each tissue lesion type (no BDD, acute BDD, chronic BDD). **(A–C)** IHC for CFB. **(A)** No BDD. Labeling of lymphocytes and plasma cells at the dermal-epidermal junction (×20), with insert confirming identification of labeled mononuclear cells (×100). **(B)** Acute BDD. Intense labeling of the most superficial keratinocytes (×20), with insert showing intense cytoplasmic and membranous labeling of the most superficial keratinocytes (×x400). **(C)** Chronic BDD lesion (×20, ×400) exhibiting similar findings to **(B)**. **(D–F)** IHC for RND1. **(D)** No BDD. Multifocally, the stratum corneum has granular background labeling of the stratum corneum keratinocytes (×20, ×100). **(E)** Acute BDD. Multifocally, there are intensely labeled keratinocytes near to ulcerated regions (×20), with insert highlighting view of intense labeling (×400). **(F)** Chronic BDD. Multifocal mild granular labeling of the stratum corneum keratinocytes similar to **(D)**; however, note most of the thickened stratum corneum is not labeled (20). Insert shows background labeling of some stratum corneum keratinocytes (400).

**Figure 8:**
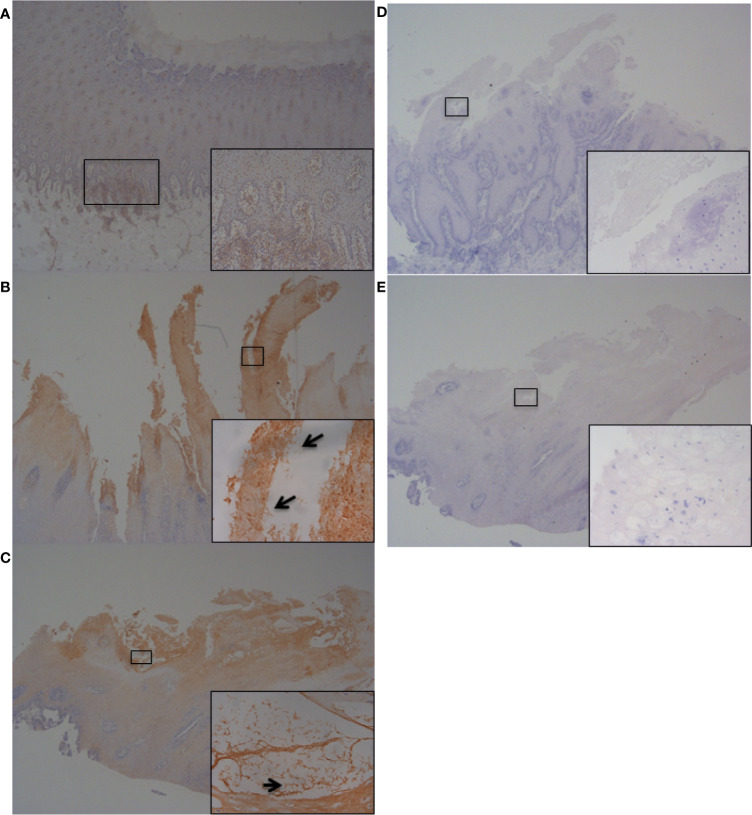
Immunohistochemistry (IHC) of IL-6 on bovine foot skin tissue from the site of BDD lesions. Low power images accompanied with high power inserts. Images shown are representatives of three independent experimental replicates (n = 3) for each tissue lesion type (no BDD, acute BDD, chronic BDD). **(A–C)** IHC for IL-6. **(A)** No BDD. Lymphocytes and plasma cells at the dermal–epidermal junction are intensely labelled (×20, ×100). **(B)** Acute BDD. Positive labeling of the stratum corneum and some stratum spinosum keratinocytes (×40), with insert showing intense labeling of spirochaetal structures (×400). Arrow denotes labeling showing similar morphology and location to that observed with DD treponeme IHC (**Figure S4E**) **(C)** Chronic BDD lesion (×20, ×400) exhibiting similar findings to **(B)**. Arrow denotes labeling showing similar morphology and location to that observed with DD treponeme IHC (**Figure S4F**). **(D–E)** IL-6 negative antibody (no primary antibody) control. **(D)** Acute BDD (×20, ×400). **(E)** Chronic BDD (×20, ×400).

To further investigate and validate findings of increased abundance in *CFB*, *RND1*, *IL-6* and *IL-8* transcripts in fibroblasts challenged with three BDD treponemes *in vitro*, IHC was performed to detect the encoded proteins within bovine foot skin tissues from cows with acute (M2) and chronic (M4) BDD lesions and those without (M0) (**Figures 7** and **8**). Matched histology was also performed to identify key features of each tissue for comparison (**Figure S4**). While CFB was expressed moderately within keratinocytes of the stratum basale and deep stratum spinosum in foot tissues with no BDD lesions (**Figure 7A**), it was intensely expressed within superficial keratinocytes in feet of both acute and chronic BDD lesions (**Figures 7B, C** **)**. RND1 expression was detected in the superficial stratum corneum keratinocytes of tissues with acute (M2) BDD lesions (**Figure 7E**) when compared with tissues without lesions (**Figure 7D**). An increase in RND1 expression was not detected in the chronic BDD lesions assessed here (**Figure 7F**). Tissues with both acute and chronic BDD lesions had increased expression of IL-6 within the superficial stratum corneum (**Figures 8B, C**). Interestingly, in these sections, spirochaetal bacteria were also labeled with IL-6 (**Figures 8B, C** inserts), as confirmed by complimentary labeling with an anti-DD treponeme antibody (**Figures S4D–F**) and appropriate negative antibody controls (**Figures 8D, E** **)**. IL-8 expression differed between the tissues of acute and chronic BDD lesions and those without lesions. While IL-8 was mostly expressed at the dermal–epidermal junction in infiltrating leukocytes (both cytoplasmic and membraneous labeling) in tissues with acute BDD lesions, its expression was observed as multifocal, granular, cytoplasmic labeling of keratinocytes in the stratum basale and stratum spinosum in tissues with chronic BDD lesions (data not shown).

## Discussion

As a severe infectious disease of cattle, BDD is now a global animal health and welfare threat where treatment is generally ineffective. An improved understanding of pathogenic mechanisms should enable improved treatment and control. RNA-Seq has become an increasingly valuable tool for studying the underlying host–pathogen interactions of disease pathogenesis. To-date, most gene expression studies on BDD pathogenesis have focused on targeted gene approaches (Refaai et al., 2013; Evans et al., 2014). Here, we used RNA-Seq to investigate global transcriptional changes in a specific bovine foot skin cell population following challenge with treponemes to elucidate key mechanisms of BDD pathogenesis and the individual role of three key BDD *Treponema* phylogroups. Four of the highly dysregulated mRNA transcripts, *IL-8, IL-6, RND1*, and *CFB*, were further investigated for presence of encoded proteins *in vivo* in bovine foot skin tissues to confirm model relevance by demonstrating involvement in BDD lesions.

A consistent and important finding was that different phylogroups of BDD treponemes induced very distinct patterns of differential mRNA expression in bovine foot skin fibroblasts. Comparatively, *T. medium* phylogroup was much less stimulatory compared to equivalent concentrations of *T. phagedenis* and *T. pedis*. Despite maintaining inflammatory potential, *T. medium* did not induce the same dysregulation of transcripts associated with apoptosis and actin rearrangement exhibited for other spirochaetes. Previous studies identified this phylogroup as a weaker stimulant of certain inflammatory mediators (Evans et al., 2014) and less potent inhibitor of fibroblast proliferation (Boehringer et al., 1984). Notably, the *in vitro* growth requirements for *T. medium* are very distinct from other BDD treponemes which may underpin such differences (Evans et al., 2008; Evans et al., 2009b). The unique increase in *TSC22D3* transcript abundance by *T. medium* phylogroup suggests anti-inflammatory and/or immunosuppressive potential; the human *TSC22D3* gene encodes a glucocorticoid-induced leucine zipper protein known to inhibit the activation of key inflammatory mediators such as *NF-kB* (Ayroldi et al., 2001; Mittelstadt and Ashwell, 2001; Berrebi et al., 2003; Eddleston et al., 2007). It is likely that *T. medium* phylogroup spirochaetes may facilitate immune evasion, prolonged survival and persistence deep within bovine foot skin tissues through upregulating host expression of such anti-inflammatory genes. Most interestingly, the commensal treponeme, *T. ruminis*, promoted the largest inflammatory response of the treponemes, suggesting that BDD treponemes have evolved a unique adaptation in line with syphilis pathogen, *Treponema pallidum*, to avoid host detection and should therefore also be considered ‘stealth pathogens’ (Radolf et al., 2016).

Interestingly, several mRNA transcripts were found to be uniquely expressed in bovine fibroblasts following stimulation by only one *Treponema* phylogroup. For instance, only *T. medium* induced significant expression of soluble pattern recognition receptor, *PTX3*. While previously reported as upregulated in dermal fibroblasts following *B. burgdorferi* challenge (Meddeb et al., 2016), lack of *PTX3* stimulation by *T. phagedenis* and *T. pedis* is interesting, suggesting inhibition of acute phase response by these two phylogroups. Similarly, *T. phagedenis* stimulated expression of an antimicrobial peptide (beta defensin) precursor, *DEFB123*. Interestingly, treponemes exhibit resistance to beta defensins (Brissette et al., 2004) and such upregulation by *T. phagedenis* spirochaetes would be advantageous to all treponemes and explain their dominance within BDD lesions. The transcript abundance of pro-inflammatory cytokine, interleukin 36A, was uniquely increased by *T. pedis* and, again, suggests a distinct role in pathogenesis for this spirochaete.

As an inflammatory disease, BDD is associated with an abundance of infiltrating immune cells, particularly neutrophils, to dermal and epidermal tissues (Blowey and Sharp, 1988; Döpfer et al., 1997; Refaai et al., 2013). Here we demonstrate the underlying mechanism for this cell migration is likely the transcription of numerous pro-inflammatory cytokines (*IL-6, IL-8*) and both monocyte (*CCL2*) and neutrophil (*CXCL2, CXCL3*, and *CXCL5*) chemoattractants which were increased in bovine foot skin fibroblasts challenged with BDD treponemes. A similar profile was observed following challenge with the commensal treponeme and *S.* Typhimurium, and in previous studies with BDD lesion tissues (Scholey et al., 2013) and fibroblasts challenged with other pathogenic spirochaetes (Nixon et al., 2000). This is indicative of a typical host response to Gram-negative bacterial LPS (Skovbjerg et al., 2010) despite treponemes reportedly exhibiting “atypical” LPS. This response appeared to be mediated through *IL-17* signaling, which has a known role in defense against Gram-negative bacteria at barrier tissues such as skin (Hymowitz et al., 2001; Ishigame et al., 2009). That disease-associated *IL-17* signaling pathways were only significantly enriched in fibroblasts following pathogenic challenge supports previous findings for a role in inflammatory skin pathologies (Infante-Duarte et al., 2000), including psoriasis (Krueger et al., 2012), spirochaete-associated Lyme arthritis and syphilis (Burchill et al., 2003; Cruz et al., 2012). Collectively, this points to a likely role for IL-17 in BDD pathogenesis and may offer treatment options, given IL-17 is a promising target for human psoriasis therapeutics (Krueger et al., 2012; Leonardi et al., 2012).

Abundance of several other mRNA transcripts was significantly increased in fibroblasts following challenge by each treatment group, including those associated with inhibition of NF-*k*B signaling (*Mail, TNFSF15*), activation of the alternative pathway of the complement cascade (*CFB*), protection from cellular oxidative stress (*SOD2*), and nucleotide metabolism (*CDA*) (Nygaard, 1986; Skalerič et al., 2000; Janeway et al., 2001). *SOD2* upregulation has been reported in human gingival fibroblasts following challenge with the pathogenic spirochaete, *B. burgdorferi* (Schramm et al., 2012). Notably, until now, BDD treponemes have yet to be implicated in the dysregulation of complement components, such as classical component C2 dysregulated by *T. medium* spirochaetes here.

Using our *in vitro* skin model of BDD pathogenesis, bovine fibroblasts were found to increase transcript abundance of an inflammatory cytokine, *IL-8*, following challenge by all three BDD treponemes. *In vivo* IHC experiments performed here confirmed previous reports that host skin tissue keratinocytes have increased IL-8 expression in BDD lesions (Refaai et al., 2013; Scholey et al., 2013). IL-8 has a recognized role in neutrophil chemotaxis during the host immune response (Caswell et al., 1999; Nixon et al., 2000) and has been consistently implicated in BDD pathogenesis and that of other spirochaete-associated pathologies including Lyme disease (Marchal et al., 2009). *T. phagedenis* phylogroup spirochaetes have been shown to induce *IL-8* upregulation in bovine macrophages (Zuerner et al., 2007). Our study suggests that bovine fibroblasts may also be implicated in upregulating IL-8 expression in BDD lesions.

This study highlights several mRNA transcripts, including *RGS16, GRO1, MAFF*, and *ZC3H12A*, which were only stimulated by BDD treponemes (and *S.* Typhimurium); however, they have yet to be implicated in BDD pathogenesis. *CXCL1*, the orthologous human *GRO1* gene, was previously found to be significantly upregulated in dermal fibroblasts and chondrocytes following challenge with *B. burgdorferi* and its protease, BbHtrA, respectively (Schramm et al., 2012; Russell et al., 2013). MAFF is a transcriptional regulator of inflammatory and cellular stress responses through its association with cap ‘n’ collar basic region-leucine zipper (bZIP) transcription factors (Ishii et al., 2000; Motohashi and Yamamoto, 2004; Massrieh et al., 2006). ZC3H12A, encoding MCP-induced protein 1, is an RNase inhibitor of inflammatory mRNA targets such as IL-6 and IL-17 (Koga et al., 2011; Garg et al., 2015) whose dysregulation is associated with inflammatory psoriasis (Guatam et al., 2011) and *B. burgdorferi* challenge (Ruiz-Romeu et al., 2016). RGS16 is a GTPase-activating protein which negatively regulates G protein-coupled receptor signalling pathways and can modulate immune cell chemotaxis and inhibit production of monocyte-derived pro-inflammatory cytokines such as IL-8, IL-6 and TNF-alpha (Lippert et al., 2003; Shankar et al., 2012; Suurväli et al., 2015). Again, RGS16 dysregulation has been linked to chronic inflammatory skin pathologies including psoriasis and atopic dermatitis (Li et al., 2013).

The actin cytoskeleton is essential for maintaining cellular morphology, tissue integrity and facilitating fibroblast migration during wound healing and tissue repair (Tojkander et al., 2012). It is a known target of pathogenesis for Gram-negative bacteria, including treponemes (Baehni et al., 1992; Yang et al., 1998; Fullner and Mekalanos, 2000; Zuerner et al., 2007). *Treponema denticola* reduces filamentous actin expression, increases rearrangement of stress fibers and causes detachment of human gingival fibroblasts (Baehni et al., 1992; Yang et al., 1998; Visser et al., 2011). BDD treponemes also appear to trigger such mechanisms, including downregulation of cytoskeletal genes (*ACTN1*, cytoskeletal-associated protein 1) in bovine macrophages following *T. phagedenis* stimulation (Zuerner et al., 2007). In the present study, both *T. medium* phylogroup and *T. pedis* dysregulated transcription associated with actin rearrangement and a loss of adhesion in bovine foot skin fibroblasts. *T. pedis* significantly inhibited actin and cytoskeleton signaling pathways and reduced organization of the cytoskeleton, cytoplasm, and microtubules with reduced quantities of focal adhesions, stress fibers and actin filaments. The transcription of several F-actin-associated proteins (*ACTN1, WIP2*, *SYNPO, NEXN*), a Rho family GTPase, *RHOG*, a regulator of wound healing, *FHL3*, and an integral component of actin polymerisation, *CFL1*, each significantly decreased in fibroblasts following treatment with *T. pedis* (Gauthier-Rouviére et al., 1998; Aspenström, 2002; Maciver and Hussey, 2002; Wang et al., 2005; Zimin et al., 2009). Most interestingly, both *T. medium* phylogroup and *T. pedis* increased *RND1* transcription in bovine fibroblasts. *RND1* gene upregulation is associated with cell rounding due to its inhibition of actin stress fibre formation and loss of integrin-based focal adhesions (Nobes et al., 1998). Together, our data suggests that *T. medium* and *T. pedis* spirochaetes, but notably not *T. phagedenis*, are able to compromise fibroblast cell–cell adhesion and therefore promote deeper invasion and persistence within bovine foot skin tissues. The increased RND1 protein expression identified by IHC and observed in superficial keratinocytes of bovine foot skin tissues with acute BDD lesions, compared to those without lesions, further supports a pathogenic role for RND1.

Interestingly, while *T. pedis* spirochaetes are considered most closely related to the human periodontal pathogens, *T. denticola* and *Treponema putidum* (Evans et al., 2008), it was *T. phagedenis* phylogroup spirochaetes which instead appeared to be associated with a periodontal disease phenotype. This may be due to differences in the tissue locality of *T. phagedenis* and the typically superficial location of *T. pedis* spirochaetes *in vivo.*

The IHC experiments on bovine foot skin tissues confirmed observations of increased expression of the four targets (*IL-8, IL-6, CFB*, and *RND1*) in the skin with acute and/or chronic BDD lesions, as similarly identified by the transcription studies of fibroblast cell–treponeme interactions, thereby confirming their importance in BDD pathogenesis. This further supports fibroblasts as a useful model for studying BDD pathogenesis and is supported by their wide use in disease pathogenesis studies of other spirochaetes to-date (Nixon et al., 2000; Meddeb et al., 2016). The data also supports the use of spirochaete sonicates, rather than intact live microorganisms, in such host–pathogen interaction studies. Indeed, the use of sonicated spirochaete preparations has been demonstrated for several previous studies (Zuerner et al., 2007; Evans et al., 2014), owing to the challenging growth conditions of these fasitidious anaerobes, and has been shown to produce representative expression profiles to live cell equivalents (Ebnet et al., 1997). While several molecules were investigated further, this was not possible for all identified markers due to the absence of an extensive protein detection toolkit for bovines. Interestingly, keratinocytes were the predominant cell lineage identified in the *in vivo* pathogenic changes, indicating that fibroblasts may have a role in how keratinocytes behave in BDD lesions. The transcriptomics study captures a very specific time period (6 h) within these interactions and so will predate many subsequent (>1 day) pathological changes.

This novel transcriptomics dataset provides unique insights into the distinct pathogenic mechanisms of three predominant BDD treponeme phylogroups in bovine foot skin fibroblasts and highlights both the complexities and importance of connective tissue cells in BDD pathogenesis. The means by which BDD treponemes persist and invade cattle skin, as well as survive anti-microbial host responses are identified. Protein localization strategies revealed that changes in gene expression were reflected by protein expression within BDD lesions. It is apparent that BDD treponemes have a unique adaptation to their bovine environment in acting as “stealth pathogens” and by dissecting these host–pathogen interactions here we have identified important targets for therapeutic intervention.

## Data Availability Statement

The datasets presented in this study can be found in online repositories. The names of the repository/repositories and accession number(s) can be found below: The RNA-Seq dataset generated and analysed for this study has been deposited in NCBI’s Gene Expression Omnibus (Edgar et al., 2002) and is accessible through GEO Series accession number GSE138011 (https://www.ncbi.nlm.nih.gov/geo/query/acc.cgi?acc=GSE138011).

## Ethics Statement

Animal sampling was reviewed and approved by the University of Liverpool Ethical Review Process, application number VREC137.

## Author Contributions

NE and SC were behind the experimental concept. KN, SC, and NE designed the study. KN performed experiments and analyzed all data collected, except the IHC experiment which was performed and analyzed by HC-D. KN wrote the manuscript. All authors contributed to the article and approved the submitted version.”

## Funding

Research was funded by a BBSRC Doctoral Training Partnership studentship (BB/M011186/1) awarded to KN and supervisors NE and SC, a BBSRC New Investigator Award (BB/K009443/1) awarded to NE and a University of Liverpool Technology Directorate Voucher.

## Conflict of Interest

The authors declare that the research was conducted in the absence of any commercial or financial relationships that could be construed as a potential conflict of interest.

## Footnotes

1
https://github.com/najoshi/sickle

2
http://usegalaxy.org

3
http://bioinfogp.cnb.csic.es/tools/venny/
