# Supplementary material for: Challenge of Bovine Foot Skin Fibroblasts With Digital Dermatitis Treponemes Identifies Distinct Pathogenic Mechanisms
Source: Front Cell Infect Microbiol. 2021 Jan 8;10:538591. doi: 10.3389/fcimb.2020.538591 (PMC7820575; doi:10.3389/fcimb.2020.538591)
Supplement: Supplementary file 1 [file DataSheet_1.docx]

Supplementary Material

# Supplementary Figures


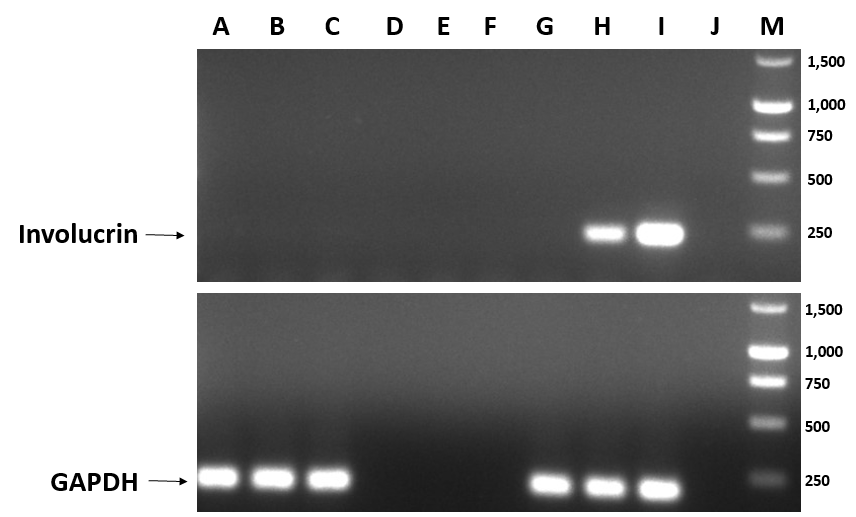


**Supplementary Figure 1.** **Detection of involucrin gene expression in primary bovine dermal foot skin cell cultures by RT-PCR to validate their fibroblast cell lineage and confirm absence of contaminating gDNA in corresponding total RNA preparations.** Gene expression of the marker for terminal differentiation in epidermal keratinocytes, involucrin, was not detected in representative dermal cell cultures from experimental replicate **(A)** one, **(B)** two or **(C)** three by RT-PCR. Expression of the housekeeping gene, glyceraldehyde 3-phosphate dehydrogenase (GAPDH), was monitored as an internal control. Products were visualised by agarose gel electrophoresis, as shown, using a 1kb DNA marker **(M)** to identify correct product sizes. **(G)** Fibroblast cell cDNA and **(J)** water were used as negative controls, whilst **(H, I)** keratinocyte cell cDNA was used as a positive control. Appropriate reverse transcription negative (RT-) controls for experimental replicate **(D)** one, **(E)** two and **(F)** three were prepared and analysed simultaneously to confirm the absence of involucrin and GAPDH expression which would otherwise suggest gDNA contamination of RNA preparations.

**
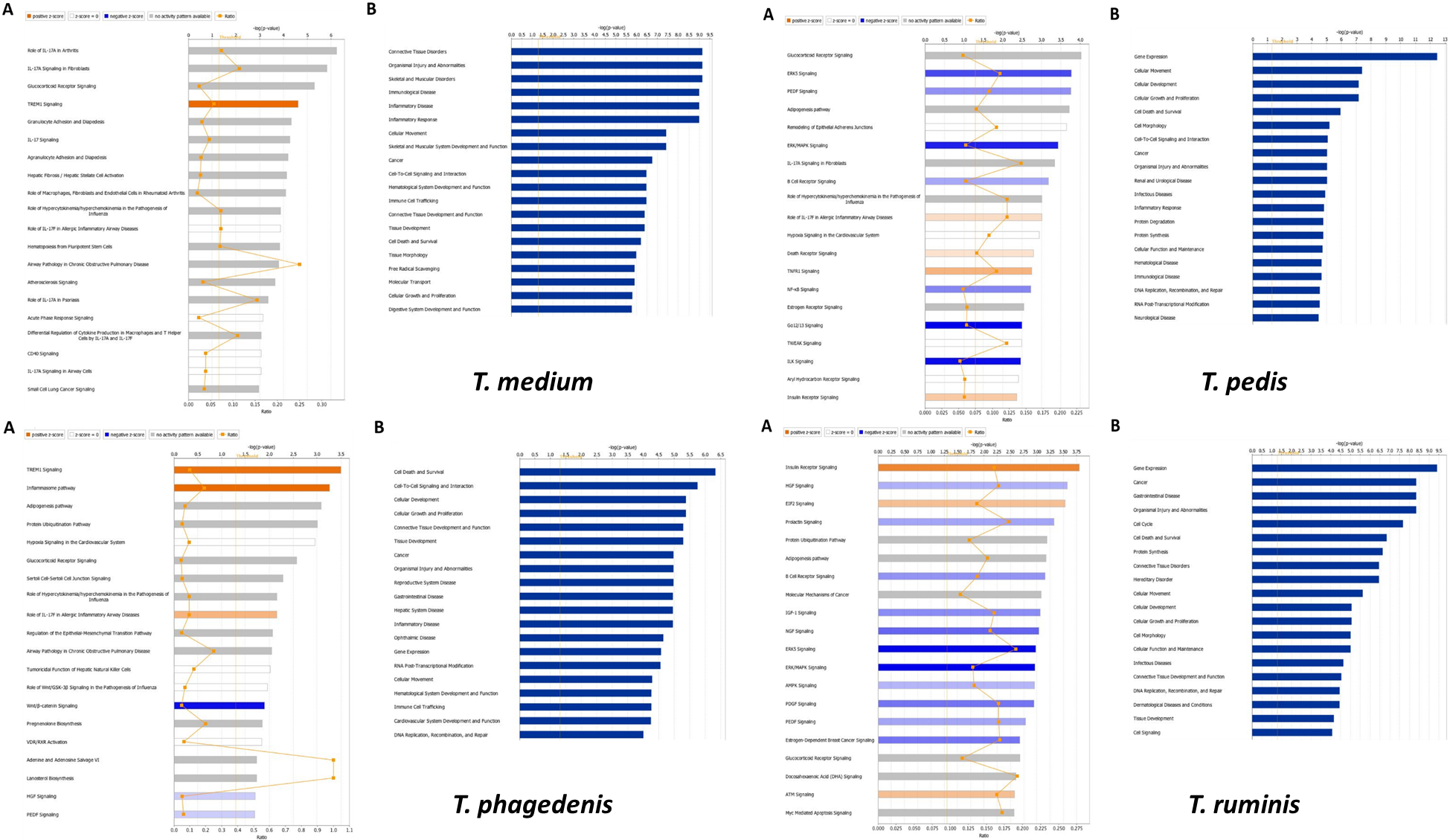
**

**Figure S2. Summary of the 20 most significantly (A) activated (orange) or inhibited (blue) canonical pathways and (B) enriched diseases and biological functions in primary bovine foot skin fibroblasts following challenge with *T. medium* (T19)*, T. phagedenis* (T320A) *or T. pedis* (T3552B) using ingenuity pathway analysis***.* Significance is depicted as bars measuring –log(p-value).


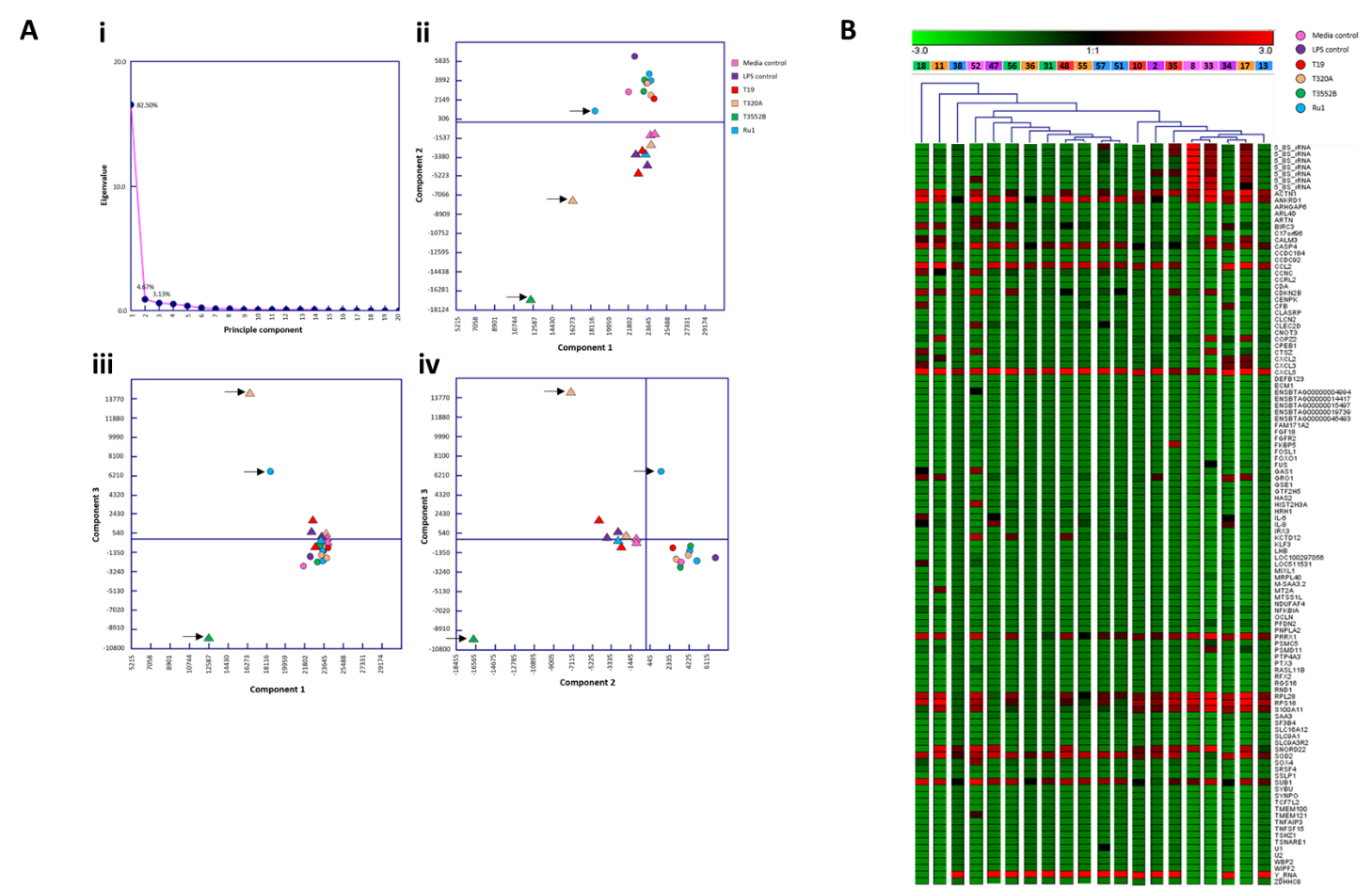


**Figure S3. Multivariate analyses of normalised mRNA expression (FPKM) from primary bovine foot skin fibroblasts challenged with pathogenic and commensal treponemes depicting the variance across all 20 experimental replicates. (A)** Principle component analysis plots illustrating **(i)** the percentage of variation explained by each of the 20 principle components, variation observed by comparison of **(ii)** principle component 1 and principle component 2, **(iii)** principle component 1 and principle component 3, **(iv)** principle component 2 and principle component 3. The circular and triangular nodes each represent two distinct variant clusters within the dataset, which correspond to the two pools of cDNA libraries processed during sequencing. Experimental replicates that were considered likely outliers are highlighted with a black arrow. **(B)** Hierarchical clustering analysis dendrograms illustrating clustering patterns and variance of normalised mRNA expression across experimental replicates for the 20 most significantly increased and decreased mRNA transcripts. Sample numbers and corresponding treatment groups (represented by different colours) are given for each replicate. Branch length directly correlates to the similarity between clusters.


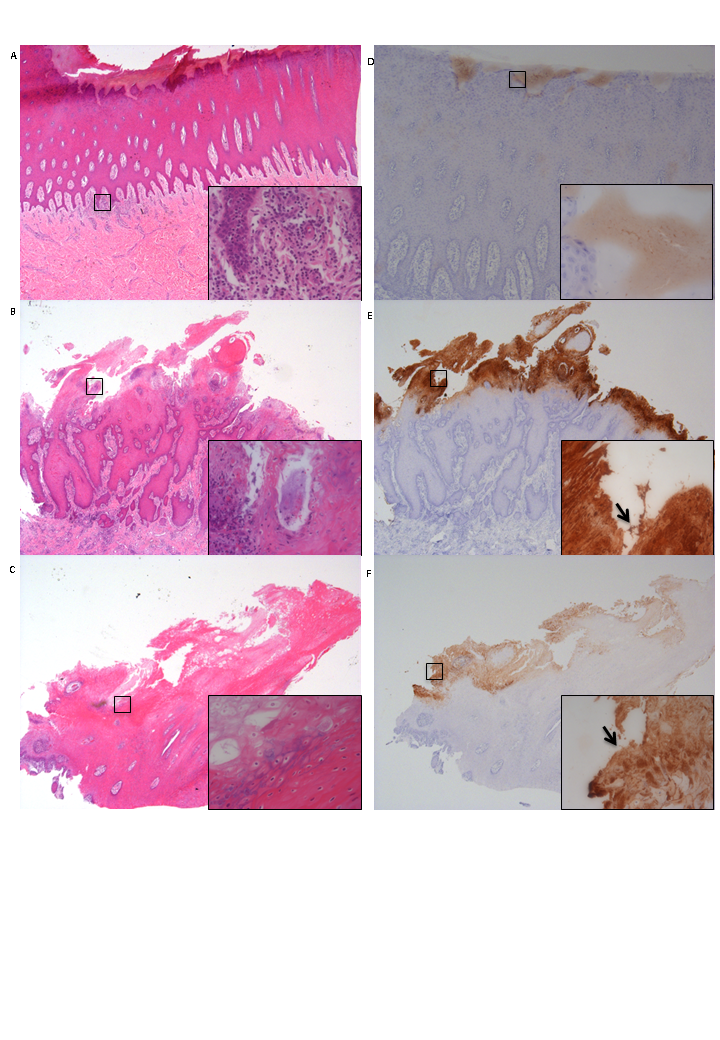


**Figure S4. Histology and additional immunohistochemistry (IHC) of bovine foot skin from the site of BDD lesions.** Low power images (x20) accompanied with high power inserts (x400). Images shown are representatives of three independent experimental replicates (n=3) for each tissue lesion type (no BDD, acute BDD, chronic BDD). **(S4A-C)** Haematoxylin and eosin stain histology sections. **(A)** No BDD. D-Dermis, SB-Stratum basale, SS-Stratum spinosum, SG-Stratum granulosum, SC-Stratum corneum. The insert shows low numbers of lymphocytes and plasma cells at the dermal-epidermal junction. Low-level, non-specific inflammation at this site is considered a background lesion in dairy cattle. **(B)** Acute BDD. Note the irregular hyperplastic epidermis with multifocal ulcers and erosion of the stratum corneum. The insert shows bacterial colonies (white arrow) and necrotic leukocytes (black arrow). **(C)** Chronic BDD. The section displays the irregular, severely thickened stratum corneum (hyperkeratosis). The insert displays bacteria tracking around keratinocytes (arrow) within a fissure. **(S4D-F)** IHC for digital dermatitis-associated treponemes. **(D)** No BDD. There is minimal faint background labelling of part of the stratum corneum. Insert showing non-specific faint labelling. **(E)** Acute BDD. The section displays a thick, intensely labelled superficial area. The insert demonstrates detection of treponeme morphology (arrow) at the periphery. **(F)** Chronic BDD. The most intense labelling is at the site where the stratum corneum is thinnest. The insert shows intense labelling at this site with treponeme morphology at the periphery.


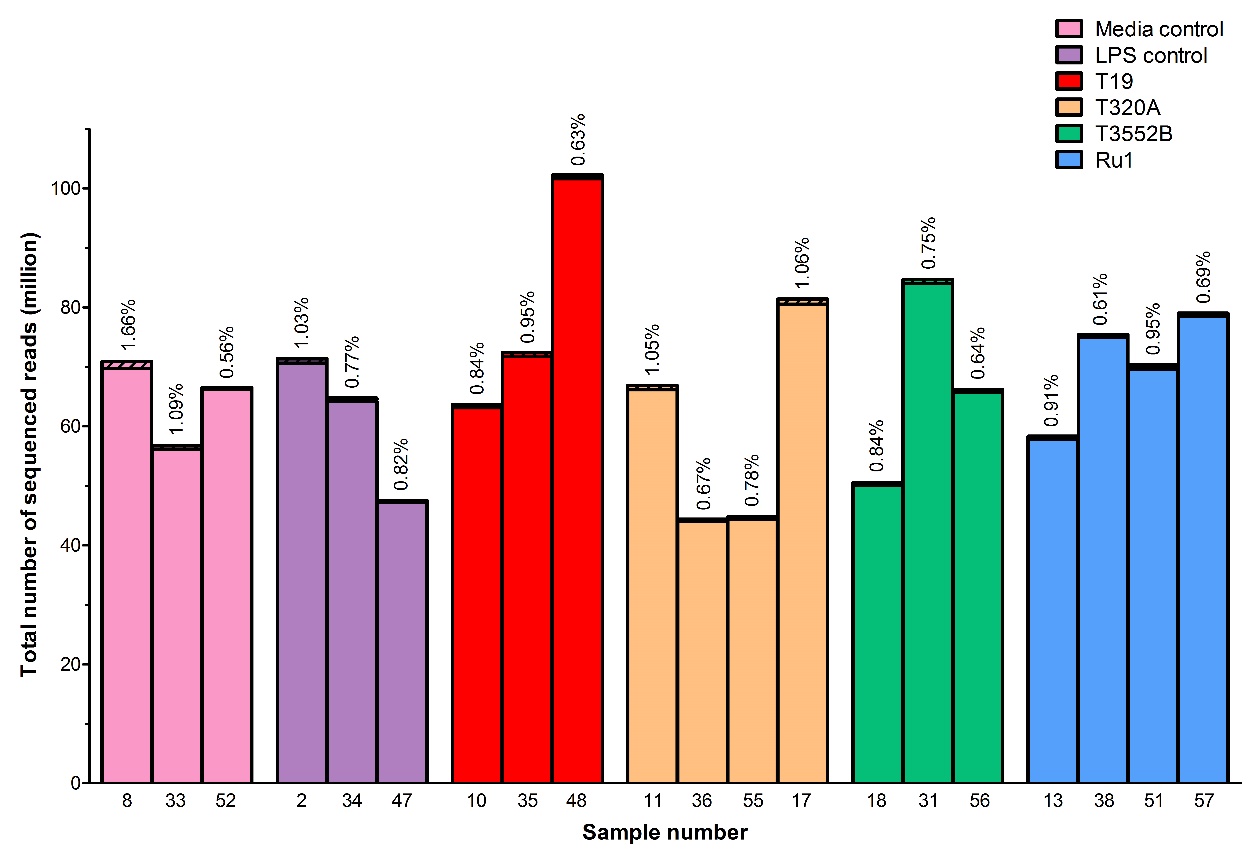


**Figure S5. The total number of sequenced reads obtained by RNA-Seq of primary bovine foot skin fibroblasts challenged with control media, *S.* Typhimurium, *T. medium, T. phagedenis, T. pedis* or *T. ruminis.*** Data is given for each challenge group for all three experimental replicates (sample numbers given), both before (plain and hashed sections) and after (plain sections) reads of poor quality were removed. The percentages of total sequenced reads excluded through quality control for each sample are given above each corresponding bar.


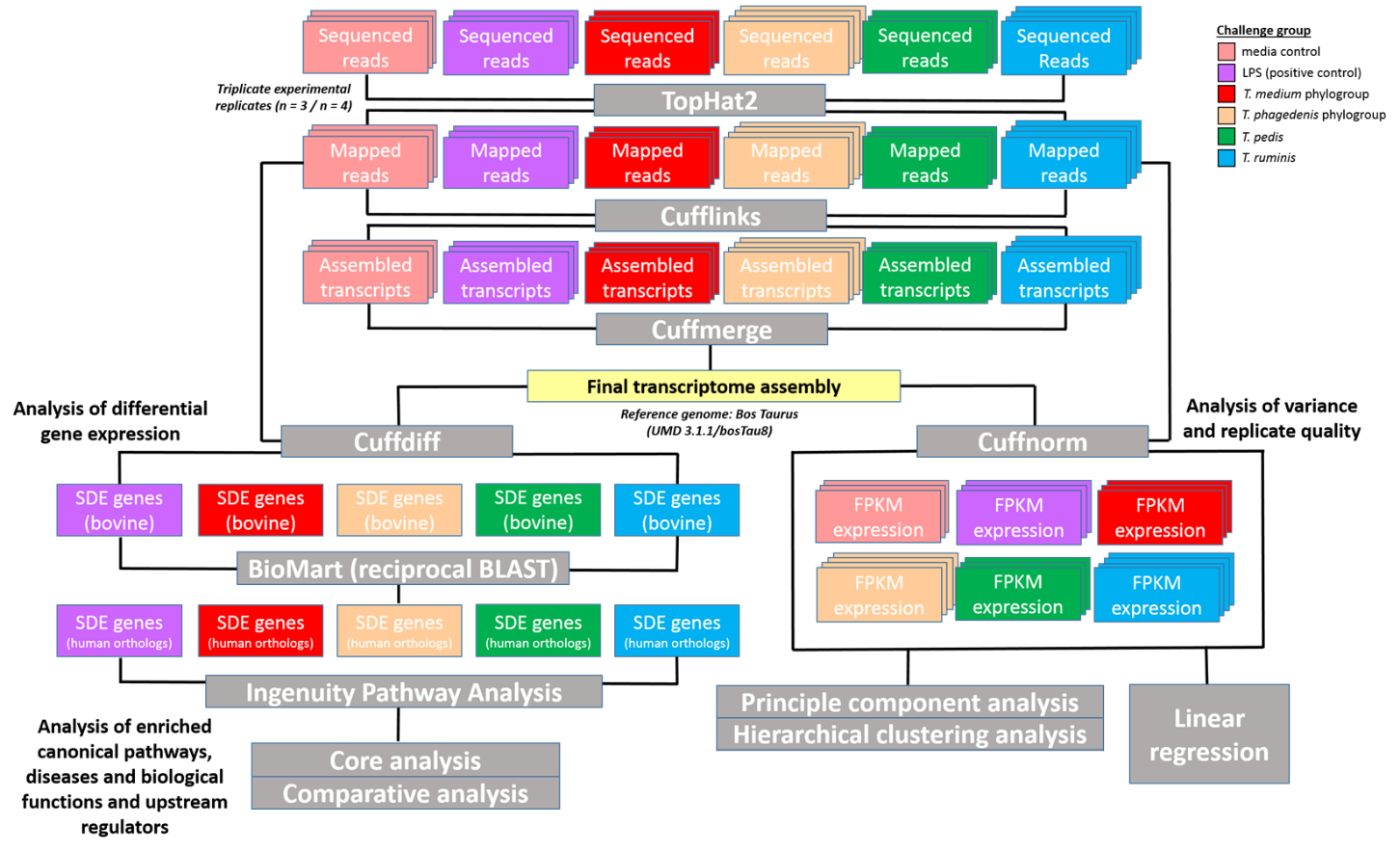


**Figure S6. RNA-Seq analysis pipeline.** RNA-Seq was performed on primary bovine foot skin fibroblasts challenged with *T. medium* phylogroup, *T. phagedenis* phylogroup, *T. pedis, T. ruminis*, *S.* Typhimurium or media (control) to allow comparative analysis of global differential mRNA transcript expression. Data represents three independent experimental replicates per challenge group (n=3), with both *T. phagedenis* and *T. ruminis* represented by an additional technical replicate (n=4). Using a pipeline modified from Trapnell’s “Tuxedo protocol” sequenced reads were mapped using TopHat2, subsequently assembled into transcripts using Cufflinks and then assembled into a final transcriptome assembly with the *Bos Taurus* reference genome [UMD 3.1.1/bosTau8] in Cuffmerge. Differential transcript abundance was analysed using Cuffdiff, whereby bovine mRNA transcripts with significantly different abundances were converted (by reciprocal BLAST in BioMart) to human orthologs to allow ingenuity pathway analysis (core and comparative). The variance of normalised mRNA expression (FPKM) across all 20 experimental replicates was analysed using Cuffnorm by principle component analysis and hierarchical clustering analysis. Linear regression was used to determine correlations in fibroblast transcript abundance following challenge by different treatment groups.

# Supplementary Tables

**Table S1. Linear regression analysis depicting correlation of transcript profiles of primary bovine foot skin fibroblasts challenged with pathogenic or commensal treponemes.** Linear regression analysis was used to determine the correlation of global differential mRNA expression, represented as log2 fold change of the 20 greatest increased and decreased transcripts between pairwise sample comparisons. The linear correlation coefficient (r), coefficient of determination (r^2^) and *p* value (p) are given, where *p* ≤ 0.05 represents statistical significance. Data represents mean differential transcript expression of three independent experimental replicates per challenge group (n=3), with both *T. phagedenis* and *T. ruminis* represented by an additional technical replicate (n=4).

| **Sample comparison** | **r value** | **r^2^ value** | ***p* value** |
| --- | --- | --- | --- |
| *S.* Typhimurium versus *T. medium* | 0.8342 | 0.6959 | <0.0001 |
| *S.* Typhimurium versus *T. phagedenis* | 0.9531 | 0.9084 | <0.0001 |
| *S.* Typhimurium versus *T. pedis* | 0.8264 | 0.6830 | <0.0001 |
| *S.* Typhimurium versus *T. ruminis* | 0.8963 | 0.8034 | <0.0001 |
| *T. medium* versus *T. phagedenis* | 0.8294 | 0.6879 | <0.0001 |
| *T. medium* versus *T. pedis* | 0.7727 | 0.5970 | <0.0001 |
| *T. medium* versus *T. ruminis* | 0.7611 | 0.5793 | <0.0001 |
| *T. phagedenis* versus *T. pedis* | 0.9032 | 0.8157 | <0.0001 |
| *T. phagedenis* versus *T. ruminis* | 0.9483 | 0.8993 | <0.0001 |
| *T. pedis* versus *T. ruminis* | 0.9192 | 0.8450 | <0.0001 |

**Table S2. Quantification and quality control of total RNA preparations from primary bovine foot skin fibroblasts challenged with control media, *S.* Typhimurium, *T. medium*, *T. phagedenis*, *T. pedis* or *T. ruminis* for RNA-Seq.**

| **Sample number** | **Challenge group** | **Experimental replicate** | **Technical replicate** | **RNA purity** | | **RNA integrity number (RIN)** | **RNA**  **yield (ng/µl)** |
| --- | --- | --- | --- | --- | --- | --- | --- |
|  |  |  |  | **A260:A280** | **A260:A230** |  |  |
| 8 | Media control | 1 | 2 | 2.05 | 1.83 | 10.00 | 99.83 |
| 2 | *S.* Typhimuirum | 1 | 1 | 2.06 | 1.83 | 10.00 | 75.93 |
| 10 | *T. medium* | 1 | 2 | 2.06 | 1.97 | 9.90 | 83.50 |
| 11 | *T. phagedenis* | 1 | 2 | 2.05 | 1.58 | 9.80 | 57.93 |
| 17 | *T. phagedenis* | 1 | 3 | 2.09 | 1.52 | 10.00 | 65.77 |
| 18^*^ | *T. pedis* | 1 | 3 | 2.08 | 1.62 | 10.00 | 56.87 |
| 13 | *T. ruminis* | 1 | 2 | 2.07 | 1.98 | 10.00 | 68.97 |
| 33 | Media control | 2 | 3 | 2.10 | 1.83 | 10.00 | 107.67 |
| 34 | *S.* Typhimuirum | 2 | 3 | 2.09 | 1.94 | 10.00 | 101.67 |
| 35 | *T. medium* | 2 | 3 | 2.08 | 1.81 | 10.00 | 68.10 |
| 36 | *T. phagedenis* | 2 | 3 | 2.08 | 2.00 | 10.00 | 79.50 |
| 31 | *T. pedis* | 2 | 2 | 2.11 | 1.92 | 10.00 | 64.20 |
| 38 | *T. ruminis* | 2 | 3 | 2.09 | 1.92 | 10.00 | 83.07 |
| 52 | Media control | 3 | 3 | 2.07 | 1.83 | 10.00 | 73.60 |
| 47 | *S.* Typhimuirum | 3 | 2 | 2.05 | 2.02 | 10.00 | 73.03 |
| 48 | *T. medium* | 3 | 2 | 2.07 | 1.98 | 10.00 | 69.17 |
| 55 | *T. phagedenis* | 3 | 3 | 2.08 | 2.03 | 10.00 | 77.80 |
| 56 | *T. pedis* | 3 | 3 | 2.07 | 2.15 | 10.00 | 67.40 |
| 51 | *T. ruminis* | 3 | 2 | 2.09 | 1.72 | 10.00 | 40.60 |
| 57 | *T. ruminis* | 3 | 3 | 2.08 | 2.08 | N/A | 84.33 |

**Required two rounds of rRNA depletion.*

**Table S3. Percentage of forward pair (R1), reverse pair (R2) and combined R1 and R2 sequenced reads successfully mapped to the *Bos taurus* genome (UMD 3.1.1/bosTau8) using TopHat2 during RNA-Seq analysis.**

| **Sample number** | **Challenge group** | **Experimental replicate** | **Technical replicate** | **Percentage of mapped reads (%)** | | **Overall read mapping rate (%)** | **Overall read mapping rate per challenge group (%)** |
| --- | --- | --- | --- | --- | --- | --- | --- |
|  |  |  |  | **R1** | **R2** |  |  |
| 8 | Media control | 1 | 2 | 88.1 | 86.3 | 87.2 | 87.2 |
| 33 | Media control | 2 | 3 | 89.9 | 88.1 | 89.0 |  |
| 52 | Media control | 3 | 3 | 85.5 | 85.3 | 85.4 |  |
| 2 | *S.* Typhimuirum | 1 | 1 | 91.0 | 88.6 | 89.8 | 88.7 |
| 34 | *S.* Typhimuirum | 2 | 3 | 91.2 | 89.4 | 90.3 |  |
| 47 | *S.* Typhimuirum | 3 | 2 | 86.2 | 85.8 | 86.0 |  |
| 10 | *T. medium* | 1 | 2 | 90.5 | 88.5 | 89.5 | 88.1 |
| 35 | *T. medium* | 2 | 3 | 89.8 | 87.4 | 88.6 |  |
| 48 | *T. medium* | 3 | 2 | 86.2 | 86.0 | 86.1 |  |
| 11 | *T. phagedenis* | 1 | 2 | 80.5 | 78.6 | 79.5 | 83.7 |
| 17 | *T. phagedenis* | 1 | 3 | 89.2 | 86.7 | 87.9 |  |
| 36 | *T. phagedenis* | 2 | 3 | 82.5 | 82.0 | 82.3 |  |
| 55 | *T. phagedenis* | 3 | 3 | 85.2 | 84.5 | 84.9 |  |
| 18 | *T. pedis* | 1 | 3 | 80.3 | 78.8 | 79.6 | 82.8 |
| 31 | *T. pedis* | 2 | 2 | 84.0 | 83.5 | 83.8 |  |
| 56 | *T. pedis* | 3 | 3 | 85.1 | 84.7 | 84.9 |  |
| 13 | *T. ruminis* | 1 | 2 | 91.2 | 89.3 | 90.3 | 85.0 |
| 38 | *T. ruminis* | 2 | 3 | 78.1 | 77.9 | 78.0 |  |
| 51 | *T. ruminis* | 3 | 2 | 86.8 | 86.3 | 86.6 |  |
| 57 | *T. ruminis* | 3 | 3 | 85.3 | 84.9 | 85.1 |  |

**Table S4. Differentially expressed mRNA transcripts from primary bovine foot skin fibroblasts challenged with *S.* Typhimurium (LPS), *T. medium* (T19), *T. phagedenis* (T320A), *T. pedis* (T3552B) or *T. ruminis* (Ru1) which mapped to multiple Ensembl stable bovine gene identifiers during RNA-Seq analysis.** Identifiers furthest to the 5’ end consistently mapped to the near-complete or complete mRNA transcript and were subsequently used for Ingenuity Pathway Analysis.

| **Transcript identifier** | **Ensembl bovine gene identifiers of mapped genes** | **Ensembl bovine gene identifier mapped furthest to the 5’ end** | **Chromosome and locus** | **Challenge group where differential expression identified** |
| --- | --- | --- | --- | --- |
| XLOC_001707 | ENSBTAG00000045492, ENSBTAG00000046668, *RNASE4* | ENSBTAG00000019612 | chr10:26423873-26445634 | LPS, T19, T320A, T3552B, Ru1 |
| XLOC_002883 | ENSBTAG00000015007, ENSBTAG00000047575 | ENSBTAG00000047575 | chr11:74496691-74590933 | LPS, T19, T320A, T3552B, Ru1 |
| XLOC_003689 | ENSBTAG00000045555, ENSBTAG00000046276 | ENSBTAG00000046276 | chr13:50362600-50400983 | LPS, T19, T320A, T3552B, Ru1 |
| XLOC_003690 | ENSBTAG00000046776, ENSBTAG00000047962 | ENSBTAG00000046776 | chr13:50362600-50400983 | LPS, T19, T320A, T3552B, Ru1 |
| XLOC_003693 | ENSBTAG00000046631, ENSBTAG00000046716, ENSBTAG00000047102, ENSBTAG00000048089 | ENSBTAG00000048089 | chr13:50362600-50400983 | LPS, T19, T320A, T3552B, Ru1 |
| XLOC_004183 | ENSBTAG00000017475, ENSBTAG00000047223 | ENSBTAG00000017475 | chr13:58010286-58049012 | LPS, T19, T320A, T3552B, Ru1 |
| XLOC_004246 | *CPNE1, RBM12* | ENSBTAG00000006955 | chr13:65507239-65543175 | LPS, T19, T320A, T3552B, Ru1 |
| XLOC_004373 | *C8orf82, LRRC24* | ENSBTAG00000046031 | chr14:1602473-1609477 | LPS, T19, T320A, T3552B, Ru1 |
| XLOC_005491 | ENSBTAG00000039307, ENSBTAG00000047406 | ENSBTAG00000047406 | chr15:6748307-6884549 | LPS, T19, T320A, T3552B, Ru1 |
| XLOC_006525 | ENSBTAG00000039728, *TMEM88B* | ENSBTAG00000039728 | chr16:52240500-52373541 | LPS, T19, T320A, T3552B, Ru1 |
| XLOC_006601 | ENSBTAG00000037813, *TOR1AIP2* | ENSBTAG00000035230 | chr16:62493957-62528817 | LPS, T19, T320A, T3552B, Ru1 |
| XLOC_006869 | ENSBTAG00000010958, ENSBTAG00000046730 | ENSBTAG00000010958 | chr17:64230115-64279571 | LPS, T19, T320A, T3552B, Ru1 |
| XLOC_007204 | ENSBTAG00000039861, *OAS2* | ENSBTAG00000014628 | chr17:63611773-63646117 | LPS, T19, T320A, T3552B, Ru1 |
| XLOC_007360 | *MON1B, SYCE1L* | ENSBTAG00000015694 | chr18:4445955-4468213 | LPS, T19, T320A, T3552B, Ru1 |
| XLOC_007520 | *CTCF,* ENSBTAG00000008860 | ENSBTAG00000008860 | chr18:35242380-35333286 | LPS, T19, T320A, T3552B, Ru1 |
| XLOC_007741 | ENSBTAG00000047345, *NECTIN2* | ENSBTAG00000015318 | chr18:52984271-53016520 | LPS, T19, T320A, T3552B, Ru1 |
| XLOC_008723 | *DPH1, OVCA2* | ENSBTAG00000009376 | chr19:23636130-23646338 | LPS, T19, T320A, T3552B, Ru1 |
| XLOC_008339 | *CEACAM20, ZNF180* | ENSBTAG00000006805 | chr18:52638276-52675994 | T19 |
| XLOC_010971 | *AK6, TAF9* | ENSBTAG00000046192 | chr20:10300552-10314075 | LPS, T19, T320A, T3552B, Ru1 |
| XLOC_011180 | ENSBTAG00000047828, *TRAPPC13* | ENSBTAG00000016900 | chr20:13856107-13891297 | LPS, T19, T320A, T3552B, Ru1 |
| XLOC_014095 | ENSBTAG00000038577, *TSC22D4* | ENSBTAG00000038577 | chr25:36614549-36623993 | LPS, T19, T320A, T3552B, Ru1 |
| XLOC_014368 | ENSBTAG00000010904, ENSBTAG00000046752 | ENSBTAG00000010904 | chr25:26681933-26689460 | LPS |
| XLOC_015151 | ENSBTAG00000016486, ENSBTAG00000047857 | ENSBTAG00000016486 | chr27:14906505-15022818 | LPS, T19, T320A, T3552B, Ru1 |
| XLOC_016145 | *BSCL2, LRRN4CL* | ENSBTAG00000010484 | chr29:41710700-41724967 | LPS, T19, T320A, T3552B, Ru1 |
| XLOC_016789 | *LRP8, MAGOH* | ENSBTAG00000000736 | chr3:93488722-93580869 | LPS, T19, T320A, T3552B, Ru1 |
| XLOC_018299 | ENSBTAG00000018909, ENSBTAG00000046308 | ENSBTAG00000018909 | chr4:67767892-68200912 | LPS, T19, T320A, T3552B, Ru1 |
| XLOC_022651 | *ALDOB, TMEM246* | ENSBTAG00000015358 | chr8:92773691-92865307 | LPS, T19, T320A, T3552B, Ru1 |
| XLOC_023775 | *ASB12, MTMR8* | ENSBTAG00000035705 | chrX:101228032-101492856 | LPS, T19, T320A, T3552B, Ru1 |
